# Risk factors for metachronous esophageal squamous cell carcinoma after endoscopic submucosal dissection: a multicenter cohort study integrating overall risk prediction and timing of recurrence

**DOI:** 10.3389/fmed.2026.1952563

**Published:** 2026-09-23

**Authors:** Gen Gui, Lu Yang, Xu Wang, Ruixue liu, Haoran Ma, Meiling Yang, Renyu Fan, Qiao Wang, Bin Sun

**Affiliations:** 1 Department of Gastroenterology, First Affiliated Hospital of Anhui Medical University, Hefei, China; 2 Anhui Provincial Key Laboratory of Digestive Disease, First Affiliated Hospital of Anhui Medical University, Hefei, China; 3 Department of Gastroenterology, Anhui Provincial Authority Hospital, Hefei, China; 4 Department of Gastroenterology, The Second Affiliated Hospital of Anhui Medical University, Hefei, China

**Keywords:** endoscopic submucosal dissection, esophageal squamous cell carcinoma, Lugol-voiding lesions, macrocytosis, metachronous recurrence, risk factors, risk stratification, white blood cell count

## Abstract

**Background:**

Endoscopic submucosal dissection (ESD) has become the standard of care for superficial esophageal squamous cell carcinoma (ESCC). Nevertheless, the persistence of field cancerization leaves patients vulnerable to metachronous lesions, underscoring an urgent need for reliable post-ESD surveillance strategies. We reasoned that a refined risk stratification approach might optimize personalized follow-up.

**Aim:**

This study therefore aimed to identify independent predictors of metachronous ESCC after curative ESD and, secondarily, to explore factors distinguishing early from late recurrence among those who relapse.

**Methods:**

We conducted a retrospective multicenter cohort study involving 1,646 patients with curative ESD for superficial ESCC across two Chinese tertiary centers between 2014 and 2024. For the primary endpoint, we employed Cox proportional hazards regression to identify variables associated with overall metachronous recurrence. In a separate exploratory analysis restricted to the 192 patients who actually developed recurrence, we applied logistic regression to examine determinants of early recurrence (defined as ≤36.7 months, the median time to recurrence) vs. late recurrence (>36.7 months).

**Results:**

During a median follow-up of 68.6 months, 192 patients (11.7%) experienced metachronous ESCC. In the primary Cox model based on a parsimonious set of 8 variables selected via LASSO regression, the strongest predictor was high-risk Lugol pattern (type C/D vs. A/B: HR = 18.868, 95% CI: 12.494–28.495), followed by elevated white blood cell count (per 1 × 10^9^/L: HR = 1.487, 95% CI: 1.369–1.615), macrocytosis (MCV ≥106 fL, HR = 1.757, 95% CI: 1.144–2.700), and tumor size (per mm: HR = 1.021, 95% CI: 1.012–1.031). RBC (HR = 0.344, 95% CI: 0.241–0.492) and BMI (HR = 0.942, 95% CI: 0.903–0.982) were protective. The parsimonious model demonstrated excellent discrimination (Harrell's C-index = 0.924, 95% CI: 0.907–0.940), with an events-per-variable ratio of 24, confirming robustness against overfitting.

**Conclusion:**

Our findings indicate that endoscopic features—particularly Lugol pattern—dominate the prediction of overall metachronous recurrence risk. The exploratory early vs. late recurrence analysis suggests that systemic inflammatory (WBC) and nutritional/metabolic (macrocytosis) parameters may modulate recurrence timing, but these findings are hypothesis-generating and require external validation before clinical application.

## 1 Introduction

Despite the generally poor prognosis of esophageal squamous cell carcinoma (ESCC) at advanced stages, early-stage lesions confined to the mucosa or superficial submucosa are highly amenable to curative endoscopic intervention. Yet, even after successful resection, the persistence of a widely exposed and carcinogen-primed mucosal field creates an enduring vulnerability to metachronous neoplasia, a concern that complicates post-treatment management. Geographically, ESCC exhibits a striking clustering within the so-called Asian esophageal cancer belt, stretching from Iran through northern and central China, where incidence rates remain among the highest worldwide (1, 2). Within China, regional, sex-related, and socioeconomic disparities further accentuate this burden, reinforcing the urgent call for locally tailored prevention and early detection policies (3). Overall 5-year survival for all-stage ESCC hovers at a discouraging 15%–25%, largely attributable to late diagnosis (4, 5). A recent systematic review and meta-analysis further confirmed that tumor-associated lymphatic vessel density serves as a reliable prognostic biomarker for esophageal cancer after radical resection (5), underscoring the importance of identifying early-stage lesions amenable to curative endoscopic treatment.

Endoscopic submucosal dissection (ESD) has therefore supplanted esophagectomy as the preferred first-line therapy for superficial ESCC, offering *en bloc* resection irrespective of lesion size, precise histopathological evaluation, and preservation of native esophageal function—all of which collectively enhance post-treatment quality of life (6–11). Meta-analytic data confirm *en bloc* and R0 resection rates above 97% and around 92%, respectively, with 5-year disease-specific survival approaching 98% (10). Nonetheless, because the organ is left intact, the residual esophageal mucosa remains exposed to ongoing carcinogenic insults, rendering patients vulnerable to metachronous ESCC.

Field cancerization, a concept originally articulated by Slaughter et al. in 1953, offers a compelling explanatory framework: chronic exposure to carcinogens preconditions a broad epithelial territory, enabling the independent emergence of multiple neoplastic foci within the same organ (12). This phenomenon is particularly conspicuous in the upper aerodigestive tract, where the esophageal and head and neck mucosae share common exposure to alcohol- and tobacco-derived carcinogens (13). Mechanistically, acetaldehyde—the primary metabolite of ethanol—is classified as a group 1 carcinogen by the International Agency for Research on Cancer (14). Genetic polymorphisms in alcohol-metabolizing enzymes, notably ALDH2 (rs671) and ADH1B (rs1229984), substantially modulate individual susceptibility to acetaldehyde accumulation and downstream mutagenesis (15, 16). Carriers of the inactive ALDH2 allele or the slow-metabolizing ADH1B1 genotype face markedly elevated ESCC risks, and multiplicative interactions with heavy drinking and smoking have been documented (17, 18). Reported incidences of metachronous ESCC after endoscopic resection vary widely-from 8.3% to 29% across cohorts (19–22)-and a recent meta-analysis estimated a pooled incidence of 20.3% (95% CI: 13.8–26.8%), with cumulative 5- and 10-year rates of approximately 12.7% and 13.7%, respectively (23). Could we, then, accurately identify those individuals at greatest risk of such late events? This question lies at the heart of our investigation.

Beyond genetic susceptibility, the dynamic risk of metachronous ESCC continues to accumulate over time. The meta-analysis by Du et al. (23) reported that the pooled incidence increases by 0.20% per additional year of follow-up, underscoring the persistent, lifelong threat. Moreover, ESCC survivors are also predisposed to second primary malignancies elsewhere, most frequently in the head and neck region, which accounts for roughly 31% of all second primaries (23, 24). This broader field-effect burden argues against a one-time risk assessment and instead calls for dynamic, sustained surveillance strategies.

Several robust risk factors have emerged from previous studies. The presence of multiple Lugol-voiding lesions (LVLs) in the background mucosa constitutes the most consistently reported predictor, with hazard ratios ranging from 3.2 to 6.5 across independent cohorts (19, 21, 25, 26). Endoscopic grade C (≥10 LVLs per view) or grade D (irregular, multiform LVLs) signals widespread epithelial dysplasia and a substantially heightened risk of subsequent carcinogenesis (27). Other established factors include heavy alcohol intake, cigarette smoking, inactive ALDH2 genotype, macrocytosis (mean corpuscular volume ≥ 106 fL), younger age at initial ESCC diagnosis, and a history of head and neck squamous cell carcinoma (28–31). Endoscopic features of the main lesion—especially spiculated or unclear margin morphology—have likewise been linked to metachronous recurrence (21, 25). Intriguingly, while these factors are often examined individually, their combined predictive value and potential interactions remain incompletely characterized.

Recent studies have refined risk stratification to some extent: Dobashi et al. (32) demonstrated a dose-dependent relationship between LVL severity and cumulative recurrence; Naito et al. (33) proposed a simplified clinical risk score integrating endoscopic and lifestyle variables; and Ishihara (34) provided a comprehensive review advocating personalized surveillance. Yet, to our knowledge, no unified analytical framework has simultaneously incorporated endoscopic, hematological, and lifestyle determinants to evaluate both the overall risk and the temporal pattern of recurrence, particularly in large Chinese multicenter cohorts. We therefore conducted a retrospective multicenter study of 1,646 patients with curative ESD for superficial ESCC, with a median follow-up of 68.6 months. Our primary aim was to identify independent predictors of metachronous recurrence using Cox proportional hazards regression, placing special emphasis on endoscopic features (Lugol pattern and margin morphology), lifestyle exposures, and hematological parameters. As a secondary, exploratory objective, we sought to examine whether these-or additional-factors could distinguish early (≤36.7 months) from late (>36.7 months) recurrence among the 192 patients who developed metachronous ESCC. By elucidating both the risk and the timing of post-ESCC carcinogenesis, we hope to inform a more nuanced, evidence-based surveillance paradigm.

## 2 Methods

### 2.1 Patients

We conducted this retrospective, multicenter cohort study at two tertiary referral institutions—the First Affiliated Hospital of Anhui Medical University (enrollment period: June 2014 to June 2024) and the Second Affiliated Hospital of Anhui Medical University (enrollment period: June 2020 to June 2024). Consecutive patients undergoing endoscopic submucosal dissection (ESD) for superficial esophageal squamous neoplasia were screened for eligibility. The study protocol received approval from the Ethics Committee of the First Affiliated Hospital of Anhui Medical University (approval No. 2026596) and complied with the principles of the Declaration of Helsinki. Given the retrospective nature of the work and our exclusive use of de-identified data derived from routine clinical practice, the committee granted a waiver of written informed consent. All patient records were anonymized prior to any analysis, and no personally identifiable information was accessed throughout the study (Figure 1).

**Figure 1:**
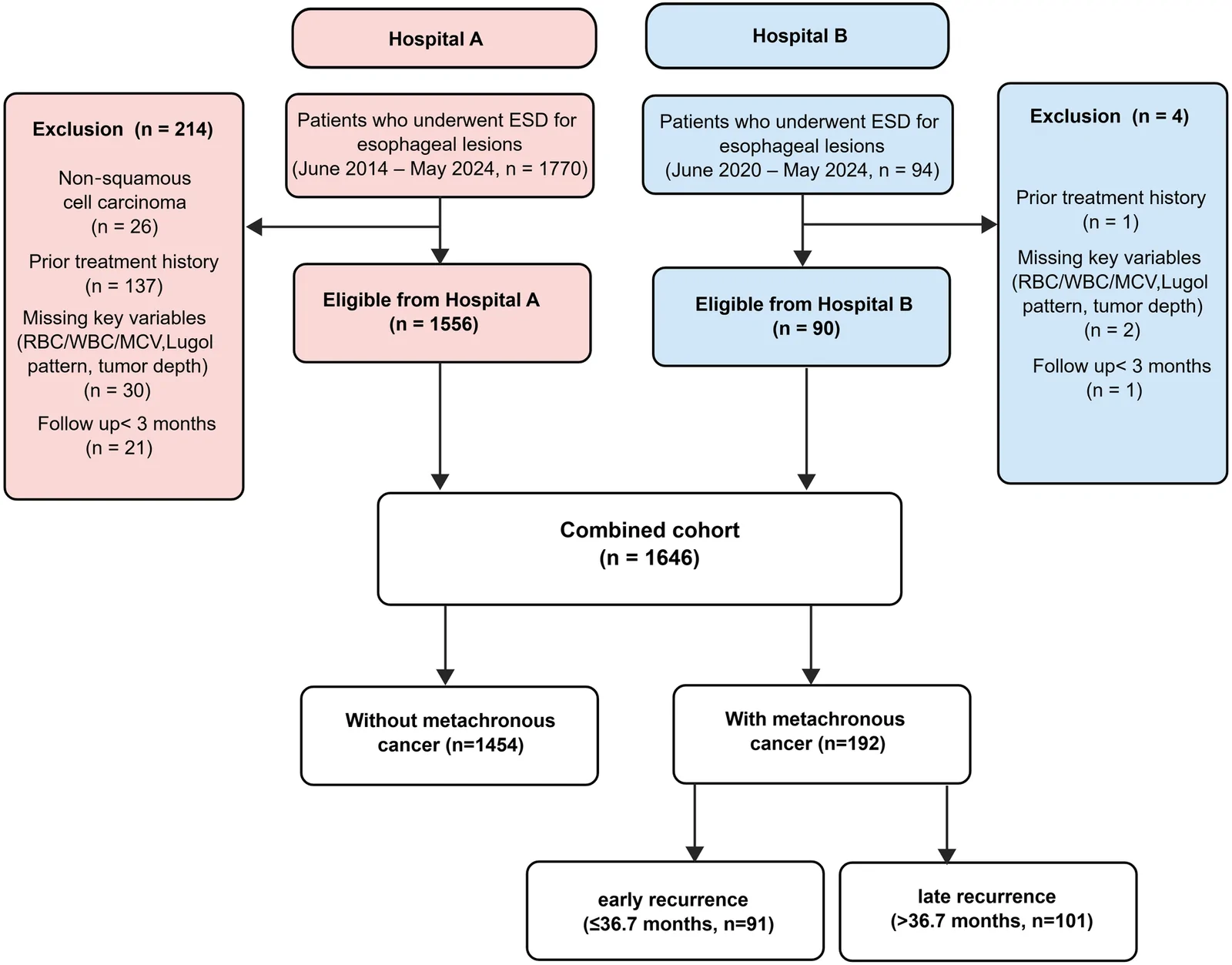
Flowchart of patient selection.

Eligibility was determined by the following inclusion criteria: (1) histologically confirmed esophageal squamous cell carcinoma (SCC) or high-grade intraepithelial neoplasia (HGIN), with preoperative staging confined to the mucosal layer (T1aN0) on the basis of endoscopic ultrasonography and computed tomography; (2) ESD performed as the primary treatment for the esophageal lesion, with no prior history of therapy for esophageal or head and neck malignancy; (3) pathological documentation of *en bloc* or R0 resection; (4) complete clinical, endoscopic, and pathological records available, including at least one complete blood count obtained within one week before ESD.

Exclusion criteria comprised: (1) a history of previous surgery, radiotherapy, or chemotherapy for esophageal or head and neck cancers; (2) receipt of additional treatment—surgery, radiotherapy, or chemoradiotherapy—within 3 months after ESD because of non-curative factors (positive resection margins, lymphovascular invasion, or deep submucosal invasion); (3) follow-up shorter than 3 months or loss to follow-up; (4) missing key variables, including age, sex, white blood cell (WBC) count, red blood cell (RBC) count, mean corpuscular volume (MCV), Lugol chromoendoscopy pattern, margin morphology, or depth of invasion.

Flowchart showing patient selection from the First Affiliated Hospital of Anhui Medical University (Hospital A, June 2014–June 2024) and the Second Affiliated Hospital of Anhui Medical University (Hospital B, June 2020–June 2024). A total of 1,770 and 94 patients were initially screened from the two centers, respectively. After exclusion, 1,646 patients were included in the final analysis, of whom 192 developed metachronous recurrence.

Exploratory subgroup definition for early vs. late recurrence: Among the 192 patients who developed metachronous recurrence, the median time from initial ESD to recurrence was calculated as 36.7 months (IQR: 25.8–53.1). Using this cohort-specific median as the cut-off, patients were stratified into an early-recurrence group (recurrence ≤36.7 months after ESD, *n* = 91) and a late-recurrence group (recurrence >36.7 months after ESD, *n* = 101) for an exploratory secondary analysis. This cut-off was empirically derived from our data and is not externally validated; therefore, all analyses based on this stratification are considered exploratory and hypothesis-generating, requiring independent validation before clinical application.

### 2.2 Data collection

Baseline demographic and clinical characteristics, including age, sex, smoking status, alcohol consumption, body mass index (BMI), and relevant medical history, were extracted from electronic medical records. Smoking was defined as either a lifetime history of at least 100 cigarettes or current daily smoking. Alcohol consumption was defined as intake of any alcoholic beverage at least once per week over a period exceeding six months.

Peripheral venous blood samples were obtained within one week prior to ESD. Routine hematological parameters, encompassing red blood cell count (RBC), white blood cell count (WBC), mean corpuscular volume (MCV), and platelet count (PLT), were measured using an automated hematology analyzer. Consistent with previous reports, macrocytosis was defined as an MCV of 106 fL or greater.

Endoscopic and pathological variables were derived from standardized procedure reports and pathology records, including tumor size (maximum diameter, mm), location (cervical/upper/middle/lower thoracic), circumferential extent (<1/4, 1/4–<1/2, 1/2–<3/4, ≥3/4), Lugol chromoendoscopy patterns (classified as type A, B, C, or D, and further simplified as low-risk A/B vs. high-risk C/D), margin morphology of the main Lugol-voiding lesion (clear, spiculated, or unclear), histological type, differentiation grade, depth of invasion, lymphovascular invasion, and resection margin status.

### 2.3 ESD procedure

All ESD procedures were performed under general anesthesia following a standardized protocol. Briefly, the lesion margin was first delineated using Lugol chromoendoscopy. Electrocautery marking spots were then placed 2–3 mm outside the margin using argon plasma coagulation (APC; ERBE, Germany) to secure an adequate cancer-free boundary. Submucosal injection of a glycerol solution containing indigo carmine and 0.0025% epinephrine was administered to lift the lesion. A circumferential incision was subsequently created with an IT knife 2 (KD-610L; Olympus Co., Ltd., Tokyo, Japan), followed by meticulous submucosal dissection. Hemostasis was achieved using hemostatic forceps (FD-410LR; Olympus) as needed, and carbon dioxide was employed for insufflation throughout the entire procedure.

En bloc resection was defined as a one-piece removal that encompassed all marking spots. R0 resection (complete resection) was defined as *en bloc* resection with histopathologically confirmed tumor-free margins.

### 2.4 Histologic assessment after ESD

Following resection, specimens were stretched, fixed on a board, and immediately immersed in 4% formalin for pathological processing. After paraffin embedding, serial sections were cut perpendicularly at 2-mm intervals and stained with hematoxylin and eosin. Histopathological evaluation encompassed tumor size, depth of invasion, lymphovascular invasion (LVI), differentiation grade, and resection margin status.

Tumor invasion depth was categorized according to the Japanese classification system for esophageal cancer, as follows: m1 (intraepithelial neoplasia), m2 (invasion of the lamina propria), m3 (invasion of the muscularis mucosae), and submucosal invasion (beyond the muscularis mucosae). For subsequent analyses, depth was dichotomized into m1 vs. deeper invasion (>m1), the latter encompassing m2, m3, and submucosal layers.

### 2.5 Follow-up of the patients

Following ESD, all patients underwent upper gastrointestinal endoscopy at 2–3 months post-procedure, primarily for exclusion of residual tumor. Thereafter, surveillance endoscopy was scheduled every 6 months during the first 3 years, and then transitioned to yearly follow-up. Lugol chromoendoscopy was routinely performed at each surveillance visit, and any Lugol-voiding areas identified were biopsied to rule out recurrent neoplasia. Annual computed tomography (CT) scans of the chest and abdomen were also undertaken to detect distant metastasis.

Metachronous recurrence was defined as histologically confirmed squamous cell carcinoma or high-grade dysplasia arising at a new location more than 6 months after ESD. The interval from the initial ESD to the detection of a metachronous event was recorded.

The surveillance schedule described above reflects the institutional practice during the study period and was implemented to ensure consistent data collection. It is not presented as a clinical recommendation derived from the present risk model. Optimal surveillance intervals based on our risk-stratification framework require prospective validation and are the subject of ongoing research. The median time from ESD to metachronous recurrence among the 192 affected patients was 36.7 months (IQR: 25.8–53.1), which was used as the cut-off for the exploratory early vs. late recurrence analysis (see Section 2.1).

### 2.6 Lugol chromoendoscopy classification

Based on the criteria proposed by Muto et al., the pattern of Lugol chromoendoscopy over the esophageal background mucosa was classified into four categories (Table 1).

**Table 1:** Classification of Lugol chromoendoscopy patterns of the esophageal background mucosa.

Type	Description
A	No Lugol-voiding lesions (LVLs)
B	Few LVLs (<10 small LVLs)
C	Multiple LVLs (≥10 small LVLs)
D	Multiple irregularly-shaped or multiform LVLs (≥10)

For clinical risk stratification, we combined A/B as “low-risk” (Lugol type A/B) and C/D as “high-risk” (Lugol type C/D). Additionally, the margin morphology of the main LVL was classified as: clear, spiculated, or unclear.

To ensure reproducibility, margin morphology was independently assessed by two experienced endoscopists (G.G. and X.W.) who were blinded to the patients' clinical outcomes. Inter-observer agreement was assessed using Cohen's *κ* statistic and was substantial (*κ* = 0.82, 95% CI: 0.76–0.88). Disagreements were resolved by consensus discussion with a third senior endoscopist (B.S.).

Endoscopic Lugol chromoendoscopy images showing different Lugol-voiding lesion patterns are presented in Figure 2. Representative images of the margin of the main Lugol-voiding lesion (clear, unclear, and spiculated) are shown in Figure 3.

**Figure 2:**
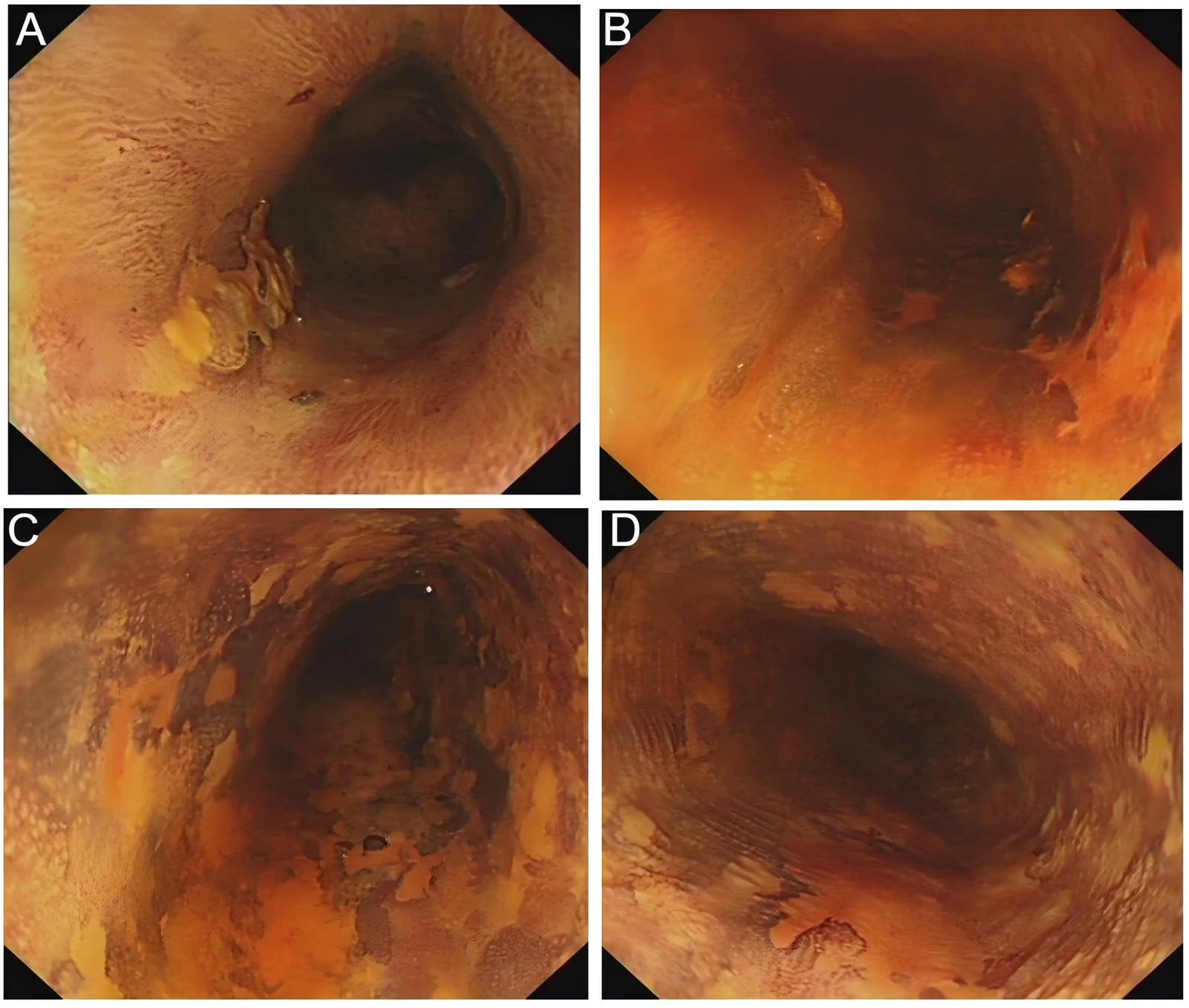
Endoscopic Lugol chromoendoscopy images. **(A)** No Lugol-voiding lesions (LVLs). **(B)** Several (≤10) small LVLs. **(C)** Many (>10) LVLs. **(D)** Many (>10) irregularly-shaped multiform LVLs around the main lesion.

**Figure 3:**
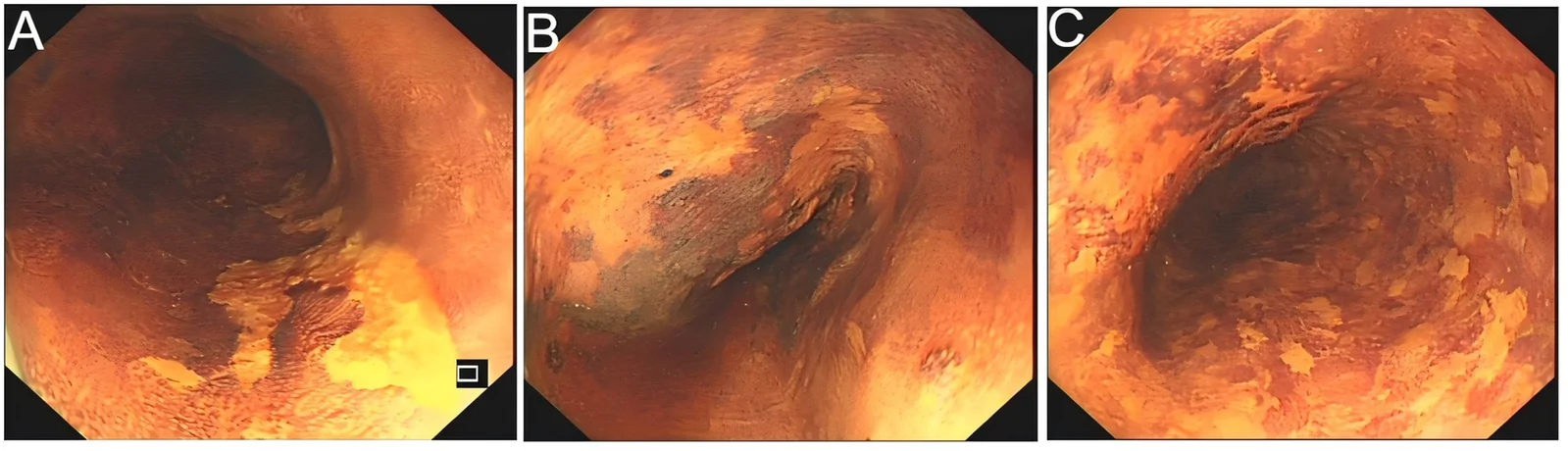
Margin of main Lugol-voiding lesion (LVL). **(A)** Clear margin. **(B)** Unclear margin. **(C)** Spiculated margin.

### 2.7 Cell culture

The human esophageal squamous cell carcinoma cell line KYSE-30 was obtained from the Cell Bank of the Chinese Academy of Sciences (Shanghai, China). Cells were maintained in RPMI-1640 medium (Gibco, Thermo Fisher Scientific, USA; 11875093) supplemented with 10% fetal bovine serum (FBS; Gibco, Thermo Fisher Scientific, USA; Cat10099141), 100 U/mL penicillin, and 100 μg/mL streptomycin (NCM Biotech, China; NCM001), at 37 °C in a humidified atmosphere containing 5% CO_2_.

The IL-6 concentration of 25 ng/mL was chosen on the basis of preliminary dose-response experiments and prior studies, which have established that this concentration effectively activates STAT3 signaling and promotes aggressive phenotypes in esophageal squamous cell carcinoma cells (21). Although this level exceeds typical circulating IL-6 concentrations (1–10 pg/mL), it recapitulates the locally elevated cytokine milieu within the tumor microenvironment, where inflammatory cells can produce IL-6 at concentrations reaching the ng/mL range.

### 2.8 Reagents

Recombinant human interleukin-6 (IL-6) was obtained from PeproTech (Cranbury, NJ, USA; 200-06). Folate-free RPMI-1640 medium was sourced from Gibco (Thermo Fisher Scientific, USA; 27016021), and dialyzed fetal bovine serum was purchased from Biological Industries (Israel; 04-011-1A).

For experimental treatments, cells were assigned to one of four groups. The control group received normal (folate-containing) RPMI-1640 supplemented with 10% FBS. The IL-6 group was exposed to 25 ng/mL IL-6 in normal RPMI-1640 for 48 h. The folate deficiency (FD) group was cultured in folate-free RPMI-1640 with 10% dialyzed FBS for the same duration. The FD + IL-6 group was maintained in folate-free RPMI-1640 containing 10% dialyzed FBS and 25 ng/mL IL-6 for 48 h. These treatment conditions, including the IL-6 concentration and exposure time, were determined based on preliminary dose-response experiments and prior reports.

### 2.9 Cell viability assay

Cell viability was evaluated using the Cell Counting Kit-8 (CCK-8; Beyotime Biotechnology, Shanghai, China; C0038). KYSE-30 cells were seeded into 96-well plates (Beyotime Biotechnology, Shanghai, China; FCP963) at a density of 5 × 10^3^ cells per well and allowed to adhere overnight. Following the designated treatments, 10 μL of CCK-8 reagent was introduced into each well, and the plates were incubated at 37 °C for 2 h. Absorbance at 450 nm was then recorded using a microplate reader (BioTek, USA). All experiments were carried out in triplicate.

### 2.10 Edu cell proliferation assay

Cell proliferation was assessed using the EdU (5-ethynyl-2′-deoxyuridine) incorporation assay kit (Beyotime Biotechnology, Shanghai, China; C0071S). KYSE-30 cells were seeded into 96-well plates (Beyotime Biotechnology, Shanghai, China; FCP963) and subjected to the designated treatments. EdU was then added to the culture medium at a final concentration of 10 μM, and cells were incubated for 2 h. Subsequently, cells were fixed with 4% paraformaldehyde, permeabilized with 0.5% Triton X-100, and stained with Apollo fluorescent dye following the supplier's recommended protocol. Nuclei were counterstained with Hoechst 33342 (Beyotime Biotechnology, Shanghai, China; C1027). Fluorescence images were captured using an Olympus microscope (Olympus, Japan), and the EdU-positive cell ratio was determined as (EdU-positive cells/Hoechst-positive cells) × 100%.

### 2.11 Wound healing assay

KYSE-30 cells were seeded into 6-well plates (Beyotime Biotechnology, Shanghai, China; FCP906) and grown to 90%–95% confluence. A straight wound was then introduced across the monolayer using a 200-µL pipette tip. After gentle washing with phosphate-buffered saline (PBS; Beyotime Biotechnology, Shanghai, China; ST476) to remove detached cells, the remaining adherent cells were subjected to the designated treatments. Phase-contrast images were captured at 0 and 48 h using an inverted microscope (Olympus, Japan). Wound closure rates were quantified as: (wound area at 0 h–wound area at 48 h)/wound area at 0 h × 100%.

### 2.12 Transwell migration and invasion assays

Cell migration was evaluated using Transwell chambers (8-μm pore size; Beyotime Biotechnology, Shanghai, China; FCH124). Briefly, KYSE-30 cells were suspended in serum-free medium and seeded into the upper chamber at a density of 2 × 10^4^ cells per well, while the lower chamber was filled with medium containing 10% FBS to serve as a chemoattractant. Following the designated treatments, cells were incubated for 24 h. Non-migrated cells on the upper surface were gently removed with a cotton swab, whereas cells that had migrated to the lower surface were fixed with methanol and stained with 0.1% crystal violet (Beyotime Biotechnology, Shanghai, China; C0121). Images were acquired, and the number of migrated cells was counted in five randomly chosen fields per well under a light microscope (Olympus, Japan).

For the invasion assay, the upper chamber was pre-coated with Matrigel (Corning, USA; 354234) prior to cell seeding. All subsequent steps were carried out in accordance with the procedures described above for the migration assay.

### 2.13 Statistical analysis

All statistical analyses were performed using R software (version 4.3.0, R Foundation for Statistical Computing, Vienna, Austria) for clinical data processing and GraphPad Prism (version 9.5) for *in vitro* experimental data analysis and graphical visualization. A two-sided *P* value of <0.05 was considered statistically significant.

Baseline characteristics comparison: Continuous variables were tested for normality using the Shapiro–Wilk test. Normally distributed variables were presented as mean ± standard deviation (SD) and compared using the independent samples *t*-test or one-way ANOVA. Non-normally distributed variables were presented as median with interquartile range (IQR) and compared using the Mann–Whitney *U* test or Kruskal–Wallis test. Categorical variables were presented as frequencies (percentages) and compared using Pearson's *χ*^2^ test or Fisher's exact test, as appropriate. Standardized mean differences (SMDs) were calculated to quantify the magnitude of group differences, with SMD >0.1 indicating a meaningful difference.

#### 2.13.1 Primary analysis: overall recurrence risk (Cox regression)

All 1,646 patients were included in this analysis. The outcome was time from ESD to metachronous recurrence (months); patients without recurrence were censored at the last follow-up or study cut-off date.

Univariate Cox regression was performed for each candidate variable to estimate the hazard ratio (HR) and 95% confidence interval (CI). Candidate predictors encompassed age, sex, smoking status, alcohol consumption, BMI, RBC, WBC, MCV (dichotomized at ≥106 fL), PLT, tumor size, tumor location, circumferential extent, Lugol-voiding lesion pattern (Lugol_binary: low-risk A/B vs. high-risk C/D), margin morphology (clear/spiculated/unclear), tumor differentiation, final pathology, tumor depth (dichotomized as m1 vs. >m1), blood vessel invasion, lymphatic invasion, and ESD resection status. Variables selected via LASSO regression with 10-fold cross-validation, along with clinically important variables (age and sex), were entered into a parsimonious multivariate Cox regression model.

Model diagnostics: The proportional hazards assumption was tested using Schoenfeld residuals (global *P* ≥ 0.05 indicating no violation). Martingale residuals were plotted to evaluate the linearity assumption for continuous variables. Deviance residuals and dfbeta values were assessed to identify influential outliers. Model discrimination was evaluated using Harrell's C-index (with 95% CI). Internal validation was performed using bootstrap resampling with 1,000 iterations to calculate the optimism-corrected C-index.

Variable selection and multicollinearity assessment: Variables with near-zero variance (e.g., final pathology, which was balanced between recurrence and non-recurrence groups with *P* = 1.000) were not entered into the multivariate model as they could not contribute predictive information. Multicollinearity among the remaining candidate variables was assessed using variance inflation factors (VIF). All VIF values were below 2.0 (mean VIF = 1.23, range: 1.02–1.89), indicating no significant collinearity among the predictors included in the final model.

Subgroup and sensitivity analyses: Subgroup analyses were performed to assess the consistency of the Lugol staining pattern effect across strata defined by sex, smoking, and alcohol drinking, with interaction terms added to the model. Sensitivity analyses included fitting a full-variable model and a reduced model (including only variables with *P* < 0.01 in univariate analysis), as well as LASSO regression with 10-fold cross-validation.

Center-stratified sensitivity analysis: To address potential confounding from different enrollment windows (Hospital A: 2014-2024; Hospital B: 2020-2024) and variations in follow-up intensity, endoscopists, and clinical standards across the two centers, we performed a center-stratified Cox regression analysis. In this sensitivity analysis, the baseline hazards were allowed to differ between centers (strata = Hospital), while the coefficients for all predictors were constrained to be equal. The hazard ratios and 95% CIs were compared with those from the primary model to assess robustness to center-specific biases. Given the small sample size from Hospital B (*n* = 90, 5.5% of the total cohort), the stratified analysis was expected to yield estimates similar to the primary model.

#### 2.13.2 Exploratory secondary analysis: early vs. late recurrence (logistic regression)

This analysis was restricted to the 192 patients who developed metachronous recurrence. Using the median time to recurrence (36.7 months) as the cut-off, patients were stratified into an early-recurrence group (≤36.7 months, *n* = 91) and a late-recurrence group (>36.7 months, *n* = 101).

Univariate logistic regression was performed for each candidate variable to estimate the odds ratio (OR) and 95% CI. Variables with *P* < 0.10 were entered into a multivariate logistic regression model using a forward stepwise selection procedure based on AIC minimization.

Important note: This secondary analysis is exploratory. The 36.7-month cut-off is cohort-specific and derived from the observed median recurrence time. Results should be interpreted as hypothesis-generating and require external validation before clinical application. Discriminative ability was quantified using the area under the receiver operating characteristic curve (AUC) via the R package pROC.

#### 2.13.3 *In vitro* experiments

All cellular assays were carried out in triplicate, with data expressed as mean ± SD. Multiple-group comparisons were evaluated using one-way ANOVA followed by Tukey's *post-hoc* test, whereas two-group comparisons were performed using Student's *t*-test (all conducted in Prism). All statistical tests were two-sided, and a *P* value <0.05 was considered statistically significant.

### 2.14 Ethics statement

This multicenter study was reviewed and approved by the Clinical Medical Research Ethics Committee of the First Affiliated Hospital of Anhui Medical University, which acted as the lead ethics committee (Approval No. 2026596). The Second Affiliated Hospital of Anhui Medical University, as a participating site, acknowledged the ethical approval granted by the lead IRB and subsequently evaluated the feasibility of conducting the study at its own facility. All procedures were performed in compliance with the principles of the Declaration of Helsinki. Given the retrospective design, the requirement for written informed consent was waived by both IRBs. Patient records were anonymized prior to analysis to safeguard privacy and confidentiality.

## 3 Results

### 3.1 Clinical features

A total of 1,646 patients were included in the final analysis, with a median follow-up of 68.6 months (IQR: 56.5–80.0). The median age was 67 years (IQR: 60–72), and the majority were male (1,195, 72.6%). During the follow-up period, 192 patients (11.7%) developed metachronous esophageal cancer, while 1,454 patients (88.3%) did not.

Compared with the non-event group, patients in the metachronous group had significantly higher white blood cell (WBC) counts (median 8.2 vs. 6.7 × 10^9^/L, *P* < 0.001, SMD=1.089), a significantly higher proportion of MCV ≥ 106 fL (14.1% vs. 3.0%, *P* < 0.001, SMD = 0.279), and significantly lower red blood cell (RBC) counts (4.6 ± 0.3 vs. 5.0 ± 0.5 × 10^12^/L, *P* < 0.001, SMD = 0.898). The proportion of current smokers was significantly higher in the metachronous group (77.6% vs. 70.4%, *P* = 0.048, SMD = 0.116). No significant differences were observed between the two groups regarding age (*P* = 0.990), sex (*P* = 0.163), alcohol drinking (*P* = 0.278), BMI (*P* = 0.333), or platelet count (*P* = 0.932). History of head and neck cancer was present in only 7 patients (0.4%) overall, all of whom were in the non-event group (0.5% vs. 0.0%, *P* = 1.000). Detailed clinical features are presented in Table 2.

**Table 2:** Clinical features of patients with and without metachronous esophageal cancer.

Characteristics	No metachronous recurrence (*n* = 1454)	Metachronous recurrence (*n* = 192)	*P* value	SMD
*Demographics*				
Age, years, median (IQR)	67 (60–72)	67 (60–72.2)	0.99	0.003
Sex, *n* (%)			0.163	0.082
Male	1,047 (72.0)	148 (77.1)		
Female	407 (28.0)	44 (22.9)		
*Lifestyle factors*				
Smoking, *n* (%)			0.048	0.116
Yes	1,024 (70.4)	149 (77.6)		
No	430 (29.6)	43 (22.4)		
Alcohol drinking, *n* (%)			0.278	0.063
Yes	425 (29.2)	64 (33.3)		
No	1,029 (70.8)	128 (66.7)		
BMI, kg/m^2^, mean ± SD	22.4 ± 3.3	22.2 ± 3.4	0.333	0.076
*Medical history*				
History of HNSCC, *n* (%)			1	0.069
Yes	7 (0.5)	0 (0.0)		
No	1,447 (99.5)	192 (100.0)		
*Hematological parameters*				
RBC, ×10^12^/L, mean ± SD	5.0 ± 0.5	4.6 ± 0.3	<0.001	0.898
WBC, ×10^9^/L, median (IQR)	6.7 (5.7–7.8)	8.2 (7.3–9.4)	<0.001	1.089
PLT, ×10^9^/L, mean ± SD	238.6 ± 50.0	238.9 ± 46.6	0.932	0.006
MCV ≥ 106 fL, *n* (%)	44 (3.0)	27 (14.1)	<0.001	0.279

HNSCC, head and neck squamous cell carcinoma; RBC, red blood cell; WBC, white blood cell; PLT, platelet; MCV, mean corpuscular volume; SMD, standardized mean difference; IQR, interquartile range; SD, standard deviation.

### 3.2 Endoscopic and pathological characteristics

The endoscopic and pathological characteristics of the study population are summarized in Table 3. Patients who developed metachronous recurrence had significantly larger tumors compared with those who did not (median 45 vs. 35 mm, *P* < 0.001, SMD = 0.568). Regarding tumor location, the majority of tumors in both groups were located in the upper or middle thoracic esophagus, with no significant difference in distribution between the two groups (*P* = 0.149). Circumferential extension showed a significant difference (*P* < 0.001), with a higher proportion of patients in the event group having 1/2–<3/4 circumferential involvement (31.2% vs. 13.8%).

**Table 3:** Endoscopic and pathological characteristics of patients with and without metachronous esophageal cancer.

Characteristics	No metachronous recurrence (*n* = 1,454)	Metachronous recurrence (*n* = 192)	*P* value	SMD
*Tumor characteristics*				
Size, mm, median (IQR)	35 (25–45)	45 (35–53.5)	<0.001	0.568
Location, *n* (%)			0.149	0.184
Cervical	52 (3.6)	3 (1.6)		
Upper thoracic	943 (64.9)	139 (72.4)		
Middle thoracic	457 (31.5)	50 (26.0)		
Lower thoracic	1 (0.1)	0 (0.0)		
Circumferential extent, *n* (%)			<0.001	0.491
<1/4	560 (38.5)	41 (21.4)		
1/4–<1/2	640 (44.0)	81 (42.2)		
1/2–<3/4	200 (13.8)	60 (31.2)		
≥3/4	52 (3.6)	10 (5.2)		
*Pathological characteristics*				
Tumor differentiation, *n* (%)			<0.001	0.502
Well	207 (14.2)	2 (1.0)		
Moderate	854 (58.7)	122 (63.5)		
Poor	393 (27.0)	68 (35.4)		
Final pathology, *n* (%)			1	0.001
HGIN	99 (6.8)	13 (6.8)		
SCC	1,355 (93.2)	179 (93.2)		
*Lugol chromoendoscopy*				
Lugol four-category, *n* (%)			<0.001	1.472
Type A (No LVLs)	426 (29.3)	4 (2.1)		
Type B (Several, ≤10)	866 (59.6)	26 (13.5)		
Type C (Many, >10)	116 (8.0)	125 (65.1)		
Type D (Irregular multiform)	46 (3.2)	37 (19.3)		
Lugol binary, *n* (%)			<0.001	1.037
Low-risk (A/B)	1,292 (88.9)	30 (15.6)		
High-risk (C/D)	162 (11.1)	162 (84.4)		
Margin morphology, *n* (%)			<0.001	1.48
Clear	1,107 (76.1)	7 (3.6)		
Spiculated	195 (13.4)	116 (60.4)		
Unclear	152 (10.5)	69 (35.9)		
Depth of invasion, *n* (%)			0.084	0.23
m1	153 (10.5)	17 (8.9)		
m2	978 (67.3)	148 (77.1)		
m3	159 (10.9)	14 (7.3)		
Mucosa	46 (3.2)	4 (2.1)		
Submucosa	118 (8.1)	9 (4.7)		
*Vascular invasion*				
Blood vessel invasion, *n* (%)			0.062	0.122
Yes	111 (7.6)	7 (3.6)		
No	1,343 (92.4)	185 (96.4)		
Lymphatic invasion, *n* (%)			<0.001	0.238
Yes	117 (8.0)	2 (1.0)		
No	1,337 (92.0)	190 (99.0)		

Regarding tumor differentiation, poorly differentiated tumors were more common in the event group (35.4% vs. 27.0%), while well-differentiated tumors were more common in the non-event group (14.2% vs. 1.0%; *P* < 0.001, SMD = 0.502). Final pathology (HGIN vs. SCC) showed no difference between groups (*P* = 1.000), as the vast majority of patients in both groups had squamous cell carcinoma (93.2% in both groups).

With respect to Lugol chromoendoscopy, the four-category classification (types A, B, C, and D) revealed a clear gradient: type A (no Lugol-voiding lesions) was dominant in the non-event group (29.3% vs. 2.1%), while type C (many LVLs, >10) and type D (irregular multiform LVLs) were overwhelmingly more frequent in the event group (65.1% and 19.3%, respectively, vs. 8.0% and 3.2% in the non-event group; *P* < 0.001, overall SMD = 1.472). When simplified into the binary Lugol classification (low-risk A/B vs. high-risk C/D), the high-risk pattern was present in 84.4% of the event group compared with only 11.1% of the non-event group (*P* < 0.001, SMD = 1.037).

Regarding margin morphology of the main Lugol-voiding lesion, spiculated and unclear margins were overwhelmingly more frequent in the event group (60.4% and 35.9%, respectively) compared with the non-event group (13.4% and 10.5%), while clear margins were dominant in the non-event group (76.1% vs. 3.6%; *P* < 0.001, SMD = 1.480). The depth of tumor invasion did not differ significantly between the two groups (*P* = 0.084). Blood vessel invasion was present in 7.6% of the non-event group vs. 3.6% of the event group (*P* = 0.062). Lymphatic invasion was significantly more frequent in the non-event group (8.0% vs. 1.0%, *P* < 0.001).

### 3.3 Short-term outcomes and complications

The short-term outcomes and procedure-related complications, stratified by metachronous event status, are presented in Table 4. En bloc resection was achieved in 90.6% (174/192) of patients in the event group compared with 96.4% (1,402/1,454) in the non-event group, with a statistically significant difference (*P* < 0.001). Patients in the metachronous group had a significantly higher proportion of non-curative factors requiring additional therapy (9.4% vs. 3.6%, *P* < 0.001), which may reflect the larger tumor size and more extensive lesions in this subgroup. ESD-related complications occurred in 2 patients (1.0%) in the event group, while no complications were observed in the non-event group (*P* = 0.014). Both complications were managed conservatively. No cases of massive bleeding, perforation, or severe stricture requiring endoscopic intervention were documented in this cohort.

**Table 4:** Short-term outcomes and complications after ESD stratified by metachronous event status.

Outcome	No metachronous recurrence (*n* = 1,454)	Metachronous recurrence (*n* = 192)	*P* value
En bloc resection, *n* (%)	1,402 (96.4)	174 (90.6)	<0.001
Non-curative factors requiring additional therapy, *n* (%)	52 (3.6)	18 (9.4)	<0.001
ESD-related complications, *n* (%)	0 (0.0)	2 (1.0)	0.014

### 3.4 Primary analysis: overall recurrence risk (Cox regression)

#### 3.4.1 Kaplan–Meier survival analysis

The overall 5-year metachronous recurrence-free survival rate was 88.2% (95% CI: 86.6–89.7%) (Figure 4A). Significant differences in recurrence-free survival were observed across multiple subgroups.

**Figure 4:**
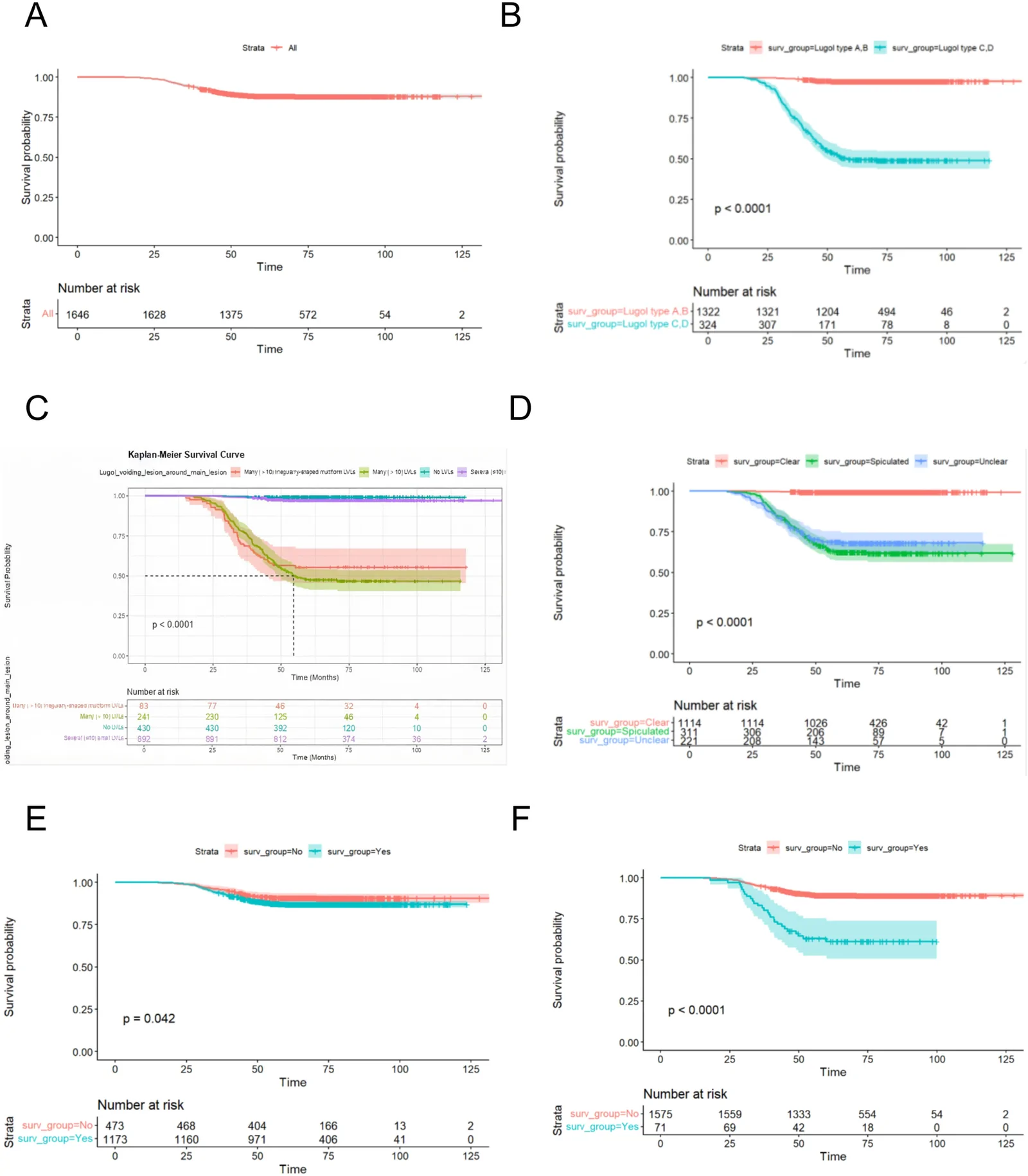
Kaplan–Meier curves for metachronous recurrence-free survival. Kaplan–Meier estimates of metachronous recurrence-free survival after endoscopic submucosal dissection (ESD). The shaded areas represent 95% confidence intervals. *P* values were calculated using the log-rank test. **(A)** Overall cohort (*N* = 1,646). The 5-year metachronous recurrence-free survival rate was 88.2% (95% CI, 86.6–89.7%). The median recurrence-free time was not reached. **(B)** Stratified by binary Lugol classification (low-risk A/B, *n* = 1,322 vs. high-risk C/D, *n* = 324). The high-risk group had a significantly higher recurrence rate (*P* < 0.001), with a median recurrence-free time of 58.5 months (95% CI: 50.1–NA). The median was not reached in the low-risk group. **(C)** Stratified by four-category Lugol classification (Type A, *n* = 430; Type B, *n* = 892; Type C, *n* = 241; Type D, *n* = 83). A stepwise increase in recurrence risk was observed from Type A to Type D (*P* < 0.001). The median recurrence-free time for Type C was 54.6 months (95% CI: 49.0–NA), while medians for Types A, B, and D were not reached. **(D)** Stratified by margin morphology (Clear, *n* = 1,114; Spiculated, *n* = 311; Unclear, *n* = 221). Both spiculated and unclear margins were associated with significantly worse recurrence-free survival compared with clear margins (*P* < 0.001). Medians were not reached for any group. **(E)** Stratified by smoking status (Non-smokers, *n* = 473; Smokers, *n* = 1,173). Smokers had a significantly higher cumulative incidence of metachronous recurrence (*P* = 0.042). Medians were not reached in either group. **(F)** Stratified by mean corpuscular volume (MCV < 106 fL, *n* = 1,575; MCV ≥ 106 fL, *n* = 71). Patients with macrocytosis (MCV ≥ 106 fL) had a significantly higher recurrence rate compared with those with MCV < 106 fL (*P* < 0.001). Medians were not reached in either group.

As shown in Figure 4B, when stratified by the binary Lugol classification (low-risk A/B vs. high-risk C/D), the high-risk group had a significantly higher cumulative incidence of metachronous recurrence (log-rank *P* < 0.001). The median recurrence-free time in the high-risk group was 58.5 months (95% CI: 50.1–NA), while the median was not reached in the low-risk group.

The four-category Lugol classification (types A, B, C, and D) is presented in Figure 4C, demonstrating a clear stepwise increase in recurrence risk from type A to type D (log-rank *P* < 0.001). Patients with type D (irregular multiform LVLs) had the highest recurrence risk, while those with type A (no LVLs) had the lowest. The median recurrence-free time for type C was 54.6 months (95% CI: 49.0–NA), while medians for types A, B, and D were not reached.

Figure 4D displays the Kaplan–Meier curves stratified by margin morphology (clear, spiculated, and unclear). Both spiculated and unclear margins were associated with significantly worse recurrence-free survival compared with clear margins (log-rank *P* < 0.001). Notably, the curves for spiculated and unclear margins were nearly overlapping, suggesting similar prognostic implications. The median recurrence-free time was not reached for any of the three groups.

Figure 4E shows the curves stratified by smoking status, where smokers exhibited a significantly higher cumulative incidence of metachronous recurrence than non-smokers (log-rank *P* = 0.042). The median recurrence-free time was not reached in either group.

Figure 4F illustrates the curves stratified by mean corpuscular volume (MCV ≥ 106 fL vs. < 106 fL). Patients with macrocytosis (MCV ≥ 106 fL) had a significantly higher recurrence rate compared with those with MCV < 106 fL (log-rank *P* < 0.001). The median recurrence-free time was not reached in either group.

Additional Kaplan–Meier analyses stratified by sex, alcohol drinking, circumferential extent, depth of invasion, and final pathology are shown in Supplementary Figures S1A–E. No significant differences were observed for sex (*P* = 0.147, Supplementary Figure S1A), alcohol drinking (*P* = 0.253, Supplementary Figure S1B), depth of invasion (*P* = 0.089, Supplementary Figure S1D), or final pathology (*P* = 0.928, Supplementary Figure S1E). However, circumferential extent showed significant differences (*P* < 0.001, Supplementary Figure S1C), with larger circumferential involvement associated with higher recurrence risk. The median recurrence-free time was not reached for any circumferential extent subgroup.

To account for potential competing risks from non-ESCC mortality, we also estimated the cumulative incidence function (CIF) for metachronous recurrence (Supplementary Figure S3). The 5-year cumulative incidence was 11.8% (95% CI: 10.2–13.3%), consistent with the Kaplan–Meier estimate of 11.8%, indicating that competing risks had negligible impact on the cumulative incidence estimation.

*The “Number at risk” tables beneath each Kaplan–Meier curve are displayed in a reduced font size due to space constraints; complete data with readable font size are available in* Supplementary Table S4*.*

#### 3.4.2 Univariate Cox regression analysis

Univariate Cox regression analysis identified multiple factors significantly associated with metachronous recurrence, as detailed in Table 5. Significant risk factors included: smoking (HR: 1.42, 95% CI: 1.01–1.99, *P* = 0.043), MCV ≥ 106 fL (HR: 4.26, 95% CI: 2.83–6.40, *P* < 0.001), WBC (per 1 × 10^9^/L, HR: 1.79, 95% CI: 1.65–1.94, *P* < 0.001), tumor size (per mm, HR: 1.03, 95% CI: 1.02–1.04, *P* < 0.001), Lugol type C/D (HR: 29.92, 95% CI: 20.25–44.20, *P* < 0.001), spiculated margin (HR: 71.77, 95% CI: 33.46–153.92, *P* < 0.001), and unclear margin (HR: 61.09, 95% CI: 28.07–132.95, *P* < 0.001). RBC was identified as a protective factor (per 1 × 10^12^/L, HR: 0.15, 95% CI: 0.11–0.21, *P* < 0.001). Age, sex, alcohol drinking, BMI, PLT, and all depth of invasion subcategories (m2, m3, Mucosa, Submucosa vs. m1) did not reach statistical significance (all *P* > 0.05).

**Table 5:** Univariate Cox regression analysis for metachronous recurrence.

Variable	HR	95% CI	*P* value
Age	1.001	0.984–1.018	0.952
Sex (Male vs. Female)	1.282	0.916–1.795	0.148
Smoking (Yes vs. No)	1.419	1.011–1.992	0.043
Alcohol drinking (Yes vs. No)	1.191	0.882–1.608	0.254
BMI	0.977	0.936–1.020	0.297
RBC	0.153	0.110–0.211	<0.001
MCV ≥ 106 fL (Yes vs. No)	4.258	2.834–6.399	<0.001
WBC	1.792	1.655–1.940	<0.001
Size	1.027	1.020–1.035	<0.001
Lugol type C/D (vs. A/B)	29.916	20.247–44.203	<0.001
Spiculated margin (vs. Clear)	71.768	33.463–153.922	<0.001
Unclear margin (vs. Clear)	61.092	28.072–132.952	<0.001
Tumor depth m2 (vs. m1)	1.341	0.812–2.215	0.252
Tumor depth m3 (vs. m1)	0.807	0.398–1.638	0.553
Tumor depth Mucosa (vs. m1)	0.779	0.262–2.316	0.653
Tumor depth Submucosa (vs. m1)	0.704	0.314–1.578	0.394

#### 3.4.3 Multivariate Cox regression analysis

Multivariate Cox regression analysis was performed using a parsimonious model with 8 variables (Age, Sex, BMI, RBC, MCV ≥ 106 fL, WBC, Size, and Lugol_binary) selected via LASSO regression with 10-fold cross-validation (Supplementary Table S1) (Table 6, Figure 5). The model had an events-per-variable ratio of 24 (192 events/8 variables), well above the recommended minimum of 10. WBC remained a strong independent risk factor, with each 1 × 10^9^/L increase associated with a 48.7% increase in risk (HR: 1.487, 95% CI: 1.369–1.615, *P* < 0.001). The high-risk Lugol pattern (type C/D vs. A/B) was associated with an approximately 19-fold increased risk (HR: 18.868, 95% CI: 12.494–28.495, *P* < 0.001). MCV ≥ 106 fL was independently associated with a 75.7% increased risk (HR: 1.757, 95% CI: 1.144–2.700, *P* = 0.010). Tumor size, with each 1 mm increase, was associated with a 2.1% increase in risk (HR: 1.021, 95% CI: 1.012–1.031, *P* < 0.001).

**Table 6:** Multivariate Cox regression analysis for metachronous recurrence (parsimonious model).

Variable	HR	95% CI	*P* value
Age (per year)	0.994	0.976–1.012	0.512
Sex (Male vs. Female)	0.725	0.511–1.028	0.071
BMI (per kg/m^2^)	0.942	0.903–0.982	0.005
RBC (per 10^12^/L)	0.344	0.241–0.492	<0.001
MCV ≥ 106 fL (Yes vs. No)	1.757	1.144–2.700	0.01
WBC (per 10^9^/L)	1.487	1.369–1.615	<0.001
Size (per mm)	1.021	1.012–1.031	<0.001
Lugol type C/D (vs. A/B)	18.868	12.494–28.495	<0.001

Model performance: Harrell's *C*-index = 0.924 (95% CI: 0.907–0.940); Likelihood ratio test *χ*^2^ = 642, df = 8, *P* < 0.001; Global Schoenfeld test *P* = 0.265.

HR, hazard ratio; CI, confidence interval; RBC, red blood cell; WBC, white blood cell; MCV, mean corpuscular volume.

**Figure 5:**
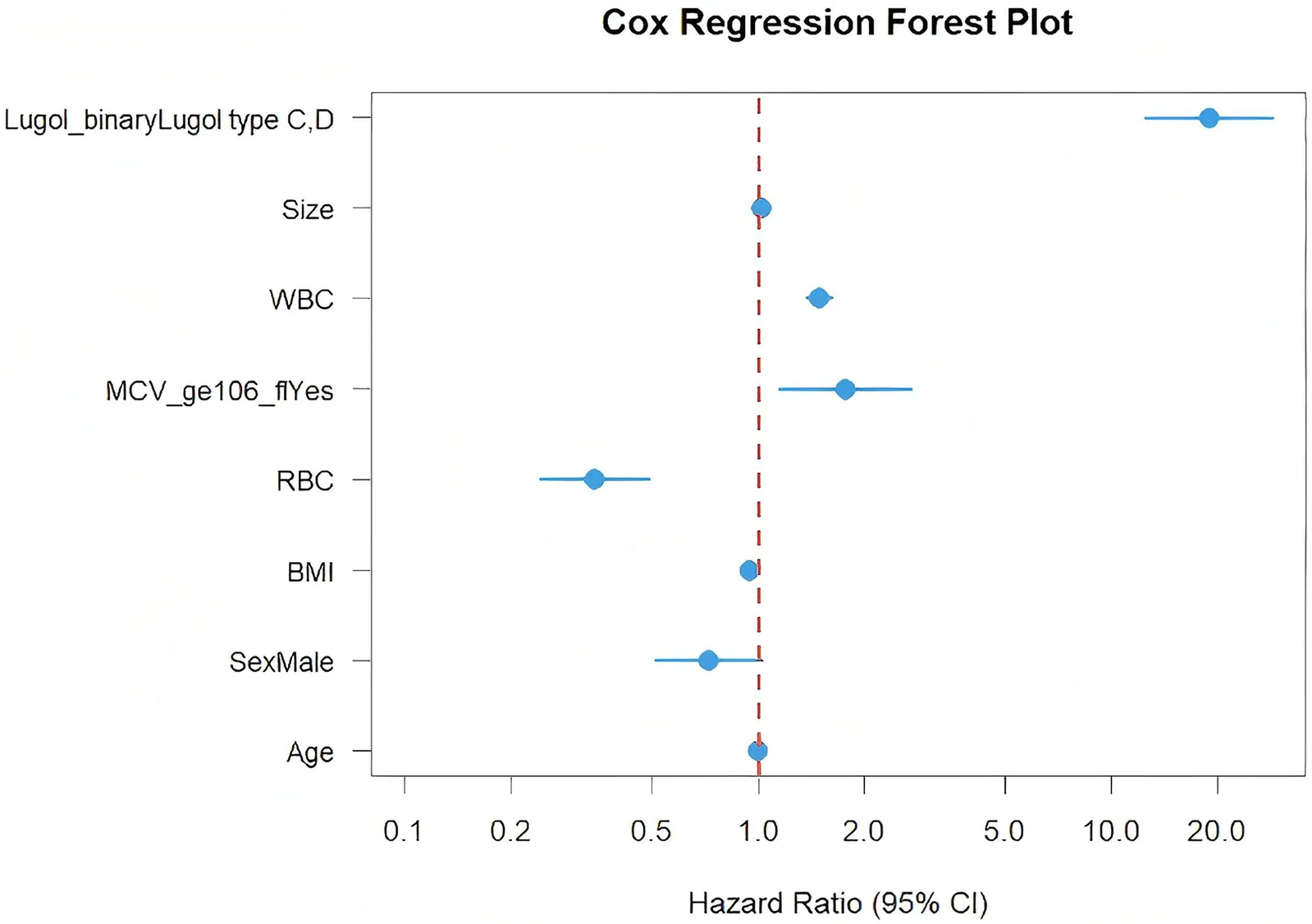
Multivariate Cox regression forest plot.

Several independent protective factors were also identified. Higher BMI was protective, with each 1 kg/m^2^ increase associated with a 5.8% risk reduction (HR: 0.942, 95% CI: 0.903–0.982, *P* = 0.005). RBC demonstrated a strong protective effect, with each 1 × 10^12^/L increase associated with a 65.6% risk reduction (HR: 0.344, 95% CI: 0.241–0.492, *P* < 0.001). Sex (Male vs. Female) showed a trend toward lower risk that did not reach statistical significance (HR: 0.725, 95% CI: 0.511–1.028, *P* = 0.071), and age was not significantly associated with recurrence risk (HR: 0.994, 95% CI: 0.976–1.012, *P* = 0.512).

The parsimonious model demonstrated excellent discrimination with a Harrell's C-index of 0.924 (95% CI: 0.907–0.940). Internal bootstrap validation with 1,000 iterations yielded an optimism-corrected C-index of 0.912, confirming the model's robustness against overfitting. The likelihood ratio test yielded *χ*^2^ = 642.02 (df = 8), *P* < 0.001, and the global Schoenfeld residual test yielded *P* = 0.265, confirming that the proportional hazards assumption was satisfied.

To account for potential bias from non-ESCC mortality as a competing event, we performed Fine-Gray subdistribution hazard regression using the same 8-variable parsimonious model (Supplementary Table S2). The subdistribution hazard ratios (sHR) were nearly identical to the cause-specific HRs from the primary Cox model, confirming that competing risks had negligible impact on our estimates.

To assess the robustness of our findings to potential center-specific biases, we performed a center-stratified Cox regression analysis (Supplementary Table S3). The hazard ratios for all core predictors remained nearly identical to the primary model: Lugol C/D (HR = 18.872, 95% CI: 12.489–28.512), WBC (HR = 1.488, 95% CI: 1.367–1.619), RBC (HR = 0.343, 95% CI: 0.240–0.490), and MCV ≥ 106 fL (HR = 1.758, 95% CI: 1.141–2.708), confirming that center-specific biases did not materially alter our estimates. The Harrell's C-index remained excellent at 0.923 (95% CI: 0.906–0.939).

We further evaluated the time-dependent predictive performance of WBC as a continuous biomarker using time-dependent ROC analysis (Supplementary Figure S4). The integrated AUC (iAUC) for WBC was 0.779, with time-specific AUCs ranging from 0.770 to 0.794, confirming its sustained predictive value throughout follow-up.

Forest plot displaying the hazard ratios (HRs) and 95% confidence intervals (CIs) for the eight variables included in the final parsimonious multivariate Cox proportional hazards model (*N* = 1,646, events = 192). Variables with *P* < 0.05 are highlighted in bold. Lugol type C/D (vs. A/B) was identified as the strongest independent risk factor, followed by WBC and MCV ≥ 106 fL. RBC and BMI were identified as independent protective factors. The dashed vertical line indicates HR = 1. Sample sizes and event counts for each variable are provided in Table 6.

#### 3.4.4 Subgroup analysis

To evaluate the robustness of the Lugol staining pattern as the primary exposure, subgroup analyses were performed stratified by sex, smoking, and alcohol drinking. The results are summarized in Table 7 and visualized in Figure 6. The high-risk Lugol pattern (type C/D vs. A/B) remained significantly associated with metachronous recurrence across all strata, with hazard ratios ranging from 14.96 to 31.16 (all *P* < 0.001, Table 7, Figure 6). Notably, a significant interaction was observed between Lugol_binary and smoking status (*P* interaction = 0.049), indicating that the effect of high-risk Lugol pattern was substantially stronger in non-smokers (HR: 31.16, 95% CI: 13.57–71.56) than in smokers (HR: 14.96, 95% CI: 9.35–23.94). However, this interaction should be interpreted with caution given its marginal statistical significance and the small number of events in the non-smoking subgroup (*n* = 43). No significant interactions were found with sex (*P* interaction = 0.081) or alcohol drinking (*P* interaction = 0.714), suggesting that the Lugol effect was consistent across these subgroups.

**Table 7:** Subgroup analysis of Lugol binary stratified by clinical factors.

Subgroup	Level	*N*	Events	HR (95% CI)	*P* value	*P* interaction
Sex	Male	1,195	148	15.81 (9.79–25.51)	<0.001	0.081
Sex	Female	451	44	30.02 (13.36–67.45)	<0.001	
Smoking	Yes	1,173	149	14.96 (9.35–23.94)	<0.001	0.049
Smoking	No	473	43	31.16 (13.57–71.56)	<0.001	
Alcohol drinking	No	1,157	128	17.25 (10.47–28.40)	<0.001	0.714
Alcohol drinking	Yes	489	64	19.92 (9.75–40.67)	<0.001	

Interpretive note: The marginal significance of the smoking interaction (P = 0.049) and the multiple subgroup comparisons performed warrant cautious interpretation; this finding requires independent confirmation.

**Figure 6:**
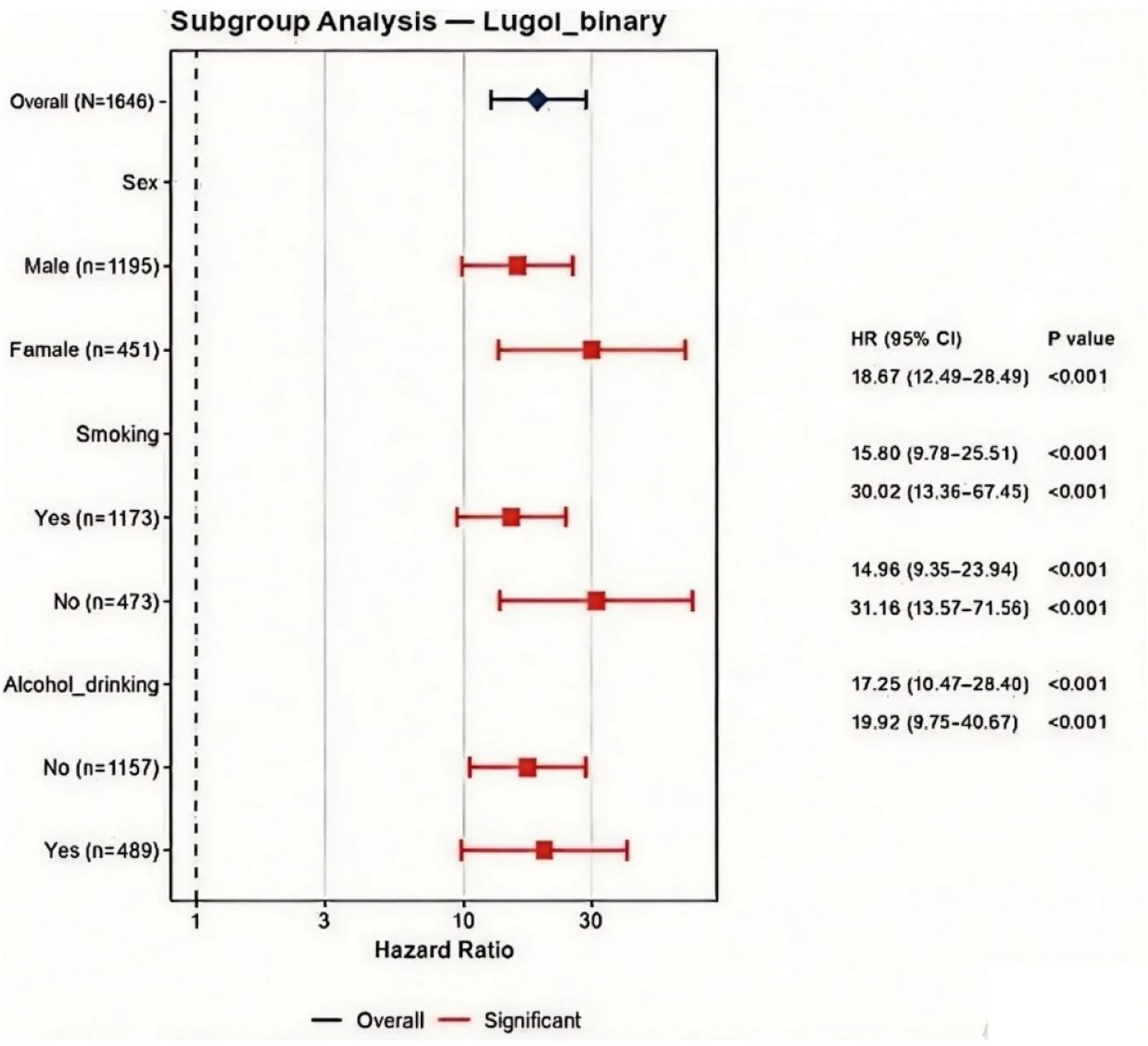
Subgroup analysis forest plot for Lugol binary.

Forest plot showing the hazard ratios (HRs) and 95% confidence intervals (CIs) for the effect of high-risk Lugol pattern (type C/D vs. A/B) on metachronous recurrence, stratified by sex (male, female), smoking (yes, no), and alcohol drinking (yes, no). The overall effect (HR: 18.87, 95% CI: 12.49–28.49) is shown at the top. *P* values for interaction are shown on the right. The high-risk Lugol pattern remained a significant risk factor across all subgroups. A significant interaction was observed with smoking status (*P*_interaction = 0.049), indicating a stronger effect in non-smokers (HR: 31.16) than in smokers (HR: 14.96). *Interpretive note: The marginal significance of this interaction (P* *=* *0.049) and the multiple subgroup comparisons performed warrant cautious interpretation; this finding requires independent confirmation.*

#### 3.4.5 Model diagnostics

The proportional hazards assumption was assessed using Schoenfeld residuals (Figure 7A). The global test yielded *P* = 0.265, and no significant temporal trends were observed for any individual covariate, confirming that the assumption was satisfied. Martingale residual plots (Figure 7B) demonstrated approximately linear relationships between continuous variables (WBC, RBC, BMI) and log-hazard, supporting the linearity assumption. Deviance residual plots (Figure 7C) revealed that the majority of residuals lay within ±2, and dfbeta values for all variables were <0.1, indicating no influential outliers that could unduly affect the model estimates. The detailed Schoenfeld residual test results for individual variables are presented in Table 8.

**Figure 7:**
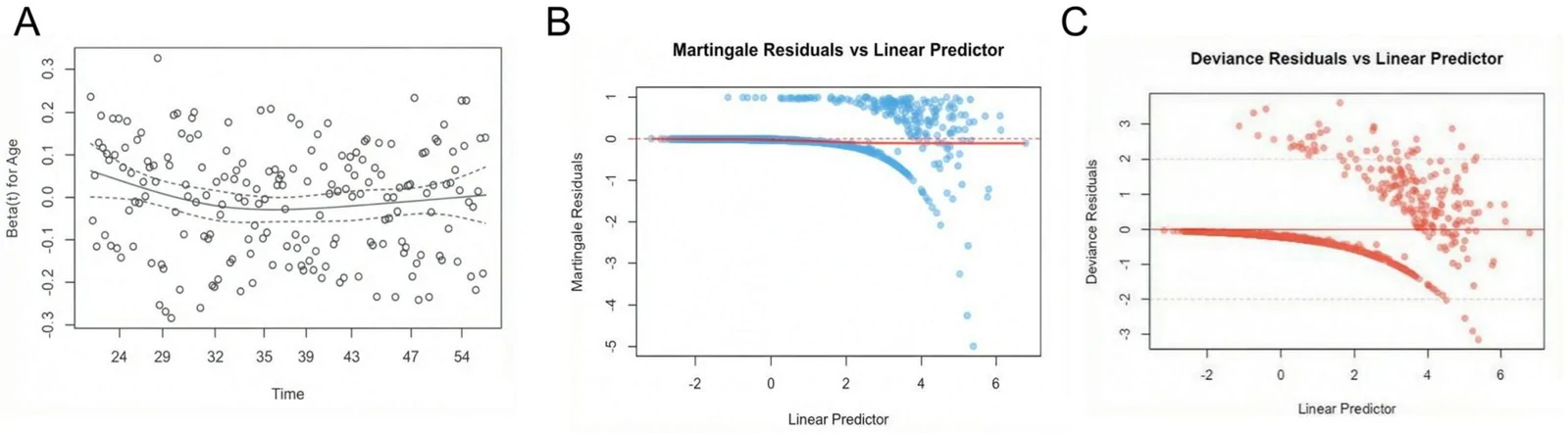
Model diagnostic plots. **(A)** Schoenfeld residual plot for Age, demonstrating no significant temporal trend (smooth line approximately horizontal), supporting the proportional hazards assumption. Similar patterns were observed for other covariates. **(B)** Martingale residual plot for WBC, showing an approximately linear relationship between WBC and log-hazard, confirming the linearity assumption. **(C)** Deviance residual plot, showing that the majority of residuals lie within ±2, with no influential outliers detected (dfbeta values < 0.1 for all variables).

**Table 8:** The detailed Schoenfeld residual test results for individual variables.

Variable	Chisq	df	*P* value
Age	1.22	1	0.27
Sex	0.014	1	0.905
BMI	1.6	1	0.206
RBC	1.41	1	0.235
MCV ≥ 106 fL	5.78	1	0.016
WBC	0.015	1	0.903
Size	0.001	1	0.98
Lugol_binary	0.942	1	0.332
GLOBAL	10	8	0.265

### 3.5 Exploratory analysis: factors associated with early vs. late metachronous recurrence

Although the primary Cox analysis identified endoscopic features as the dominant predictors of overall recurrence risk, the determinants of recurrence timing—i.e., whether a patient develops recurrence early or late—remain clinically relevant. To explore this question, we performed an exploratory secondary analysis restricted to the 192 patients who developed metachronous recurrence. Using the median time to recurrence (36.7 months, IQR: 25.8–53.1) as the cut-off, patients were stratified into early (≤36.7 months, *n* = 91) and late (>36.7 months, *n* = 101) recurrence groups.

Baseline clinical, endoscopic, and pathological characteristics of these two subgroups are presented in Table 9 (clinical features) and Table 10 (endoscopic/pathological features). Compared with patients in the late-recurrence group, those in the early-recurrence group had significantly higher WBC counts (9.2 ± 2.2 vs. 7.4 ± 1.7, *P* < 0.001) and a greater prevalence of MCV ≥ 106 fL (20.9% vs. 8.9%, *P* = 0.032). No significant intergroup differences were observed with respect to age, sex, smoking status, alcohol consumption, BMI, or RBC.

**Table 9:** Clinical characteristics of patients with early vs. late metachronous recurrence.

Characteristic	Early recurrence (*n* = 91)	Late recurrence (*n* = 101)	*P* value
Age, median [IQR]	69.0 [60.5, 73.5]	67.0 [59.0, 72.0]	0.375
BMI, mean ± SD	21.9 ± 3.0	22.4 ± 3.6	0.274
RBC, mean ± SD	4.6 ± 0.3	4.6 ± 0.3	0.961
WBC, mean ± SD	9.2 ± 2.2	7.4 ± 1.7	<0.001
PLT, mean ± SD	239.2 ± 45.8	238.6 ± 47.6	0.93
Sex, *n* (%)			0.903
Female	20 (22.0)	24 (23.8)	
Male	71 (78.0)	77 (76.2)	
Smoking, *n* (%)			1
No	20 (22.0)	23 (22.8)	
Yes	71 (78.0)	78 (77.2)	
Alcohol drinking, *n* (%)			0.959
No	60 (65.9)	68 (67.3)	
Yes	31 (34.1)	33 (32.7)	
MCV ≥ 106 fL, *n* (%)			0.032
No	72 (79.1)	92 (91.1)	
Yes	19 (20.9)	9 (8.9)	
History of head and neck cancer, *n* (%)			NA
No	91 (100.0)	101 (100.0)	

IQR, interquartile range; SD, standard deviation. This analysis is restricted to the 192 patients who developed metachronous recurrence. The 36.7-month cut-off was derived from the median recurrence time in this cohort and is not externally validated. This is an exploratory, hypothesis-generating analysis.

**Table 10:** Endoscopic and pathological characteristics.

Characteristic	Early recurrence (*n* = 91)	Late recurrence (*n* = 101)	*P* value
Size, median [IQR]	45.0 [35.0, 55.0]	45.0 [35.0, 50.0]	0.492
Location, *n* (%)			0.005
Cervical	0 (0.0)	3 (3.0)	
Middle thoracic	32 (35.2)	18 (17.8)	
Upper thoracic	59 (64.8)	80 (79.2)	
Tumor circumference, *n* (%)			0.441
<1/4	18 (19.8)	23 (22.8)	
1/4–<1/2	36 (39.6)	45 (44.6)	
1/2–<3/4	30 (33.0)	30 (29.7)	
≥3/4	7 (7.7)	3 (3.0)	
Lugol binary, *n* (%)			0.023
Lugol type A,B	10 (11.0)	25 (24.8)	
Lugol type C,D	81 (89.0)	76 (75.2)	
Margin of main LVL, *n* (%)			0.206
Clear	4 (4.4)	3 (3.0)	
Spiculated	49 (53.8)	67 (66.3)	
Unclear	38 (41.8)	31 (30.7)	
Tumor differentiation, *n* (%)			0.578
Well	1 (1.1)	1 (1.0)	
Moderate	54 (59.3)	68 (67.3)	
Poor	36 (39.6)	32 (31.7)	
Final pathology, *n* (%)			0.704
High-grade dysplasia	5 (5.5)	8 (7.9)	
Squamous cell carcinoma	86 (94.5)	93 (92.1)	
Tumor depth type, *n* (%)			0.474
m1	12 (13.2)	9 (8.9)	
>m1	79 (86.8)	92 (91.1)	

This analysis is restricted to the 192 patients who developed metachronous recurrence. The 36.7-month cut-off was derived from the median recurrence time in this cohort and is not externally validated. This is an exploratory, hypothesis-generating analysis.

Univariate logistic regression identified three factors significantly associated with early recurrence: elevated WBC (OR = 1.607 per 10^9^/L, 95% CI: 1.346–1.919, *P* < 0.001), MCV ≥ 106 fL (OR = 2.698, 95% CI: 1.152–6.317, *P* = 0.022), and Lugol type C/D (OR = 2.664, 95% CI: 1.200–5.914, *P* = 0.016) (Table 11).

**Table 11:** Univariate analysis of factors associated with early metachronous recurrence.

Variable	OR (95% CI)	*P* value
Age	1.016 (0.980–1.052)	0.389
Sex (Male vs. Female)	1.106 (0.563–2.174)	0.769
Smoking (Yes vs. No)	1.047 (0.530–2.066)	0.895
Alcohol drinking (Yes vs. No)	1.065 (0.584–1.941)	0.838
BMI	0.954 (0.876–1.039)	0.278
RBC	1.022 (0.434–2.404)	0.961
WBC (per 10^9^/L)	1.607 (1.346–1.919)	<0.001
PLT	1.001 (0.994–1.008)	0.798
MCV ≥ 106 fL	2.698 (1.152–6.317)	0.022
Size	1.007 (0.987–1.027)	0.498
Location (Middle vs. Cervical)	1.215 (0.117–12.601)	0.872
Location (Upper vs. Cervical)	0.820 (0.081–8.266)	0.866
Tumor circumference (1/4–<1/2 vs. <1/4)	0.800 (0.410–1.563)	0.514
Tumor circumference (1/2–<3/4 vs. <1/4)	0.783 (0.352–1.738)	0.547
Tumor circumference (≥3/4 vs. <1/4)	2.333 (0.551–9.889)	0.25
Lugol_binary (C/D vs. A/B)	2.664 (1.200–5.914)	0.016
Margin (Spiculated vs. Clear)	0.548 (0.117–2.578)	0.445
Margin (Unclear vs. Clear)	0.916 (0.183–4.591)	0.916
Tumor differentiation (Moderate vs. Well)	0.794 (0.049–12.983)	0.874
Tumor differentiation (Poor vs. Well)	1.125 (0.064–19.896)	0.937
Final pathology (SCC vs. HGD)	0.925 (0.291–2.941)	0.895
Tumor depth (>m1 vs. m1)	1.553 (0.622–3.877)	0.346
Blood vessel invasion (Yes vs. No)	0.826 (0.180–3.795)	0.807
Lymphatic invasion (Yes vs. No)	0.659 (0.131–3.307)	0.612
ESD resection (R0 vs. En-bloc)	0.880 (0.330–2.349)	0.798

HGD, high-grade dysplasia; SCC, squamous cell carcinoma. This is an exploratory analysis restricted to the 192 patients who developed recurrence. Results are hypothesis-generating and require external validation.

In multivariate logistic regression, both WBC (OR = 1.649, 95% CI: 1.370–1.986, *P* < 0.001) and MCV ≥ 106 fL (OR = 3.476, 95% CI: 1.349–8.952, *P* = 0.010) remained independent predictors of early recurrence, whereas Lugol_binary did not (OR = 1.782, 95% CI: 0.760–4.178, *P* = 0.184) (Table 12). The two-variable model (WBC + MCV) showed moderate discriminative ability (AUC = 0.767, 95% CI: 0.70–0.83) in distinguishing early from late recurrence (Supplementary Figure S2).

**Table 12:** Multivariate logistic regression analysis of factors associated with early metachronous recurrence.

Model	Variable	OR (95% CI)	*P* value
Two-variable model			
	MCV ≥ 106 fL	3.476 (1.349–8.952)	0.01
	WBC (per 10^9^/L)	1.649 (1.370–1.986)	<0.001
Three-variable model			
	MCV ≥ 106 fL	3.309 (1.280–8.555)	0.014
	WBC (per 10^9^/L)	1.618 (1.343–1.949)	<0.001
	Lugol_binary (C/D vs. A/B)	1.782 (0.760–4.178)	0.184

This is an exploratory analysis restricted to the 192 patients who developed recurrence. The 36.7-month cut-off is cohort-specific. Results are hypothesis-generating and require external validation before clinical application.

Important limitations of this exploratory analysis: (1) The 36.7-month cut-off was derived from the observed median recurrence time in this specific cohort and is not clinically predefined or externally validated; (2) the analysis included only 192 patients with 91 events, limiting statistical power and precluding extensive adjustment for multiple confounders; (3) results should be considered hypothesis-generating rather than confirmatory. These findings require independent validation before any clinical inferences regarding recurrence timing can be drawn.

### 3.6 *In vitro* experimental results

To preliminarily explore potential biological mechanisms that might underlie our clinical observation that elevated WBC (reflecting systemic inflammation) and macrocytosis (reflecting nutritional/metabolic disturbance) independently predict early metachronous recurrence, we examined the effects of IL-6 (used to mimic an inflammatory state) and folate deficiency (used to model macrocytosis-related metabolic abnormality) on KYSE-30 esophageal squamous cell carcinoma cells. A series of functional assays were performed to evaluate cell viability, proliferation, migration, and invasion, with the aim of determining whether these factors directly promote aggressive phenotypes in esophageal cancer cells. The experimental design is illustrated in Figure 8A, and all quantitative results are summarized in Figure 8.

**Figure 8:**
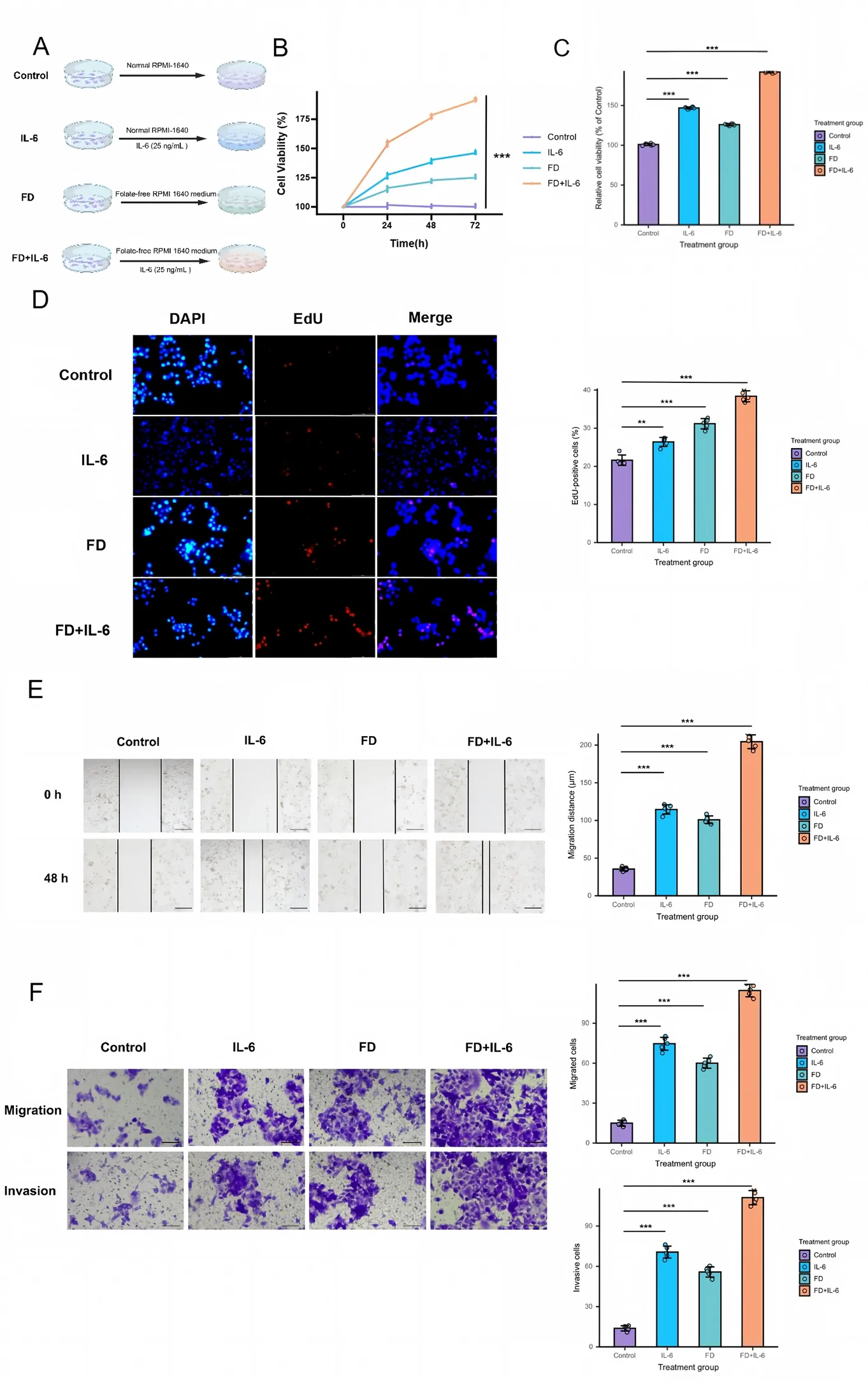
*In vitro* experimental results. **(A)** Schematic diagram showing the experimental design. KYSE-30 cells were divided into four groups: Control (normal RPMI-1640), IL-6 (25 ng/mL IL-6), FD (Folate-free RPMI 1640 medium), and FD + IL-6 (Folate-free RPMI 1640 medium + 25 ng/mL IL-6). After treatment, cell viability (CCK-8), proliferation (EdU), migration (wound healing and Transwell), and invasion (Matrigel-coated Transwell) were assessed. **(B)** CCK-8 assay time-course. Cell viability was measured at 24, 48, and 72 h. Data are presented as mean ± SD (*n* = 6). *P < 0.05, **P < 0.01, ***P < 0.001 vs. control at the corresponding time point. **(C)** CCK-8 assay at 72 h. Quantification of cell viability normalized to the control group. Data are presented as mean ± SD (*n* = 6). *P < 0.05, **P < 0.01, ***P < 0.001 vs. control. **(D)** EdU proliferation assay. Representative fluorescence images (red, EdU-positive cells; blue, Hoechst-stained nuclei) and quantification of EdU-positive cell ratio. Scale bar: 200 μm. Data are presented as mean ± SD from three independent experiments. *P < 0.05, **P < 0.01, ***P < 0.001 vs. control. **(E)** Wound healing assay. Representative images at 0 and 48 h and quantification of wound closure rate. Scale bar: 500 μm. Data are presented as mean ± SD from three independent experiments. *P < 0.05, **P < 0.01, ***P < 0.001 vs. control. **(F)** Transwell migration and invasion assays. Representative images of migrated and invasive cells stained with crystal violet, with quantification of cells per field. Scale bar: 200 μm. Data are presented as mean ± SD from three independent experiments. *P < 0.05, **P < 0.01, ***P < 0.001 vs. control. Ctrl, normal RPMI-1640; IL-6, IL-6 treatment (25 ng/mL); FD, Folate-free RPMI 1640 medium; FD + IL-6, Folate-free RPMI 1640 medium with IL-6 (25 ng/mL). Ctrl, normal RPMI-1640; IL-6, IL-6 treatment (25 ng/mL); FD, Folate-free RPMI 1640 medium; FD + IL-6, Folate-free RPMI 1640 medium with IL-6 (25 ng/mL).

#### 3.6.1 Cell viability

Cell viability was evaluated using CCK-8 assays following the designated treatments (Figures 8B,C). At 72 h, relative cell viability (normalized to the control group) was 100.93% ± 1.15% for the control, 146.93% ± 1.20% for the IL-6 group, 125.93% ± 1.15% for the folate deficiency (FD) group, and 192.03% ± 1.15% for the FD + IL-6 group (one-way ANOVA, *F* = 4890, *P* < 0.001; all pairwise comparisons *P* < 0.001, Tukey's *post-hoc* test). These findings indicate that IL-6 and folate deficiency act synergistically to enhance cell viability relative to either treatment alone.

#### 3.6.2 Cell proliferation

The EdU-positive cell ratio was 21.60% ± 1.38% for the control group, 26.39% ± 1.17% for the IL-6 group, 31.17% ± 1.47% for the FD group, and 38.37% ± 1.46% for the FD + IL-6 group, with a statistically significant difference among groups (one-way ANOVA, *F* = 131.7, *P* < 0.001). *Post-hoc* comparisons revealed that all treatment groups differed significantly from the control (*P* < 0.001 for each, Tukey's test). Notably, the FD + IL-6 group exhibited a markedly higher proliferation rate than both the IL-6 group (*P* < 0.001) and the FD group (*P* = 0.003), indicating a synergistic effect of the combined treatment (Figure 8D).

#### 3.6.3 Cell migration

In the wound healing assay, the migration distance at 48 h was 35.46 ± 2.38 μm for the control group, 114.60 ± 6.19 μm for the IL-6 group, 100.86 ± 5.26 μm for the FD group, and 204.48 ± 9.24 μm for the FD + IL-6 group, with significant differences detected among groups (one-way ANOVA, *F* = 679.5, *P* < 0.001; all pairwise comparisons *P* < 0.001) (Figure 8E).

Parallel Transwell migration assays corroborated these findings. The number of migrated cells per field was 15.00 ± 2.24 for the control, 74.60 ± 4.77 for the IL-6 group, 60.00 ± 3.81 for the FD group, and 114.60 ± 4.83 for the FD + IL-6 group, again with statistically significant intergroup differences (one-way ANOVA, *F* = 633.6, *P* < 0.001; all pairwise comparisons *P* < 0.001) (Figure 8F).

#### 3.6.4 Cell invasion

In the Matrigel-coated Transwell invasion assay, the number of invasive cells per field was 13.80 ± 1.92 for the control group, 70.60 ± 4.45 for the IL-6 group, 55.80 ± 3.96 for the FD group, and 111.20 ± 5.26 for the FD + IL-6 group, with a statistically significant difference across groups (one-way ANOVA, *F* = 601.2, *P* < 0.001; all pairwise comparisons *P* < 0.001). These findings collectively indicate that the interplay between inflammatory stimulation (IL-6) and nutritional/metabolic disturbance (folate deficiency) synergistically promotes the invasive potential of esophageal squamous cell carcinoma cells (Figure 8F).

## 4 Discussion

In this large multicenter cohort of 1,646 patients with superficial ESCC after curative ESD, we observed an overall metachronous ESCC incidence of 11.7% over a median follow-up of 68.6 months. In the primary Cox regression analysis using the parsimonious model (8 variables selected via LASSO), the strongest predictor was high-risk Lugol pattern (type C/D vs. A/B: HR: 18.87, 95% CI: 12.49–28.49), followed by WBC (HR: 1.487 per 10^9^/L, 95% CI: 1.369–1.615), macrocytosis (MCV ≥ 106 fL: HR: 1.757, 95% CI: 1.144–2.700), and tumor size (HR: 1.021 per mm, 95% CI: 1.012–1.031). RBC (HR: 0.344, 95% CI: 0.241–0.492) and BMI (HR: 0.942, 95% CI: 0.903–0.982) were protective. The model exhibited excellent discrimination (C-index 0.924, 95% CI: 0.907–0.940; optimism-corrected 0.912). In an exploratory secondary analysis restricted to the 192 patients who developed recurrence, elevated WBC (OR = 1.649) and macrocytosis (MCV ≥ 106 fL, OR = 3.476) were associated with earlier recurrence, whereas Lugol_binary lost significance (*P* = 0.184). These findings suggest that endoscopic features determine baseline recurrence risk, while systemic inflammation and nutritional/metabolic disturbances may influence the tempo of clinical manifestation.

### 4.1 Endoscopic features: the dominant predictors of overall risk

The predictive power of a high-risk Lugol pattern (type C/D) for metachronous ESCC, with a hazard ratio of 18.87 in our parsimonious model, is consistent with the broader literature (19, 21, 25, 35). Katada et al. (30) previously established a dose-dependent relationship between the severity of Lugol-voiding lesions and cumulative recurrence rates; our findings not only confirm this gradient but also suggest a substantially larger effect magnitude than that reported in their work. Lugol-unstained areas correspond to glycogen-depleted epithelium that frequently harbors dysplasia of variable grade (36), and Yokoyama et al. (37) provided compelling molecular evidence that esophageal epithelia from heavy drinkers with numerous LVLs contain widespread TP53-mutated clonal expansions, offering a biological rationale for the prognostic utility of this endoscopic sign. From a pragmatic standpoint, Lugol chromoendoscopy can be performed during routine surveillance without requiring additional equipment or extended procedure time, rendering it a readily accessible and cost-effective stratification tool. We acknowledge, however, that the subjective nature of Lugol classification may introduce interobserver variability, and the generalizability of our hazard estimates to lower-risk or non-Asian populations remains to be determined.

Although margin morphology of the main Lugol-voiding lesion was not retained in the final parsimonious model, its strong univariate association (spiculated: HR = 71.77; unclear: HR = 61.09, both *P* < 0.001) is consistent with previous reports (20). The substantial discrepancy between our univariate HR (≈70) and the previously reported HR (6.51) (20) may reflect differences in endoscopic assessment criteria, longer follow-up duration (68.6 vs. shorter in prior studies), and population-specific alcohol-related genotype distributions (ALDH2/ADH1B) that were not assessed in our study. These potential explanations remain speculative and require validation through standardized criteria and genotyping in future studies.

### 4.2 White blood cell count and systemic inflammation

Elevated WBC count at baseline independently predicted an increased risk of metachronous ESCC in our parsimonious model, with a hazard ratio of 1.487 per 10^9^/L (95% CI: 1.369–1.615, *P* < 0.001). Chronic low-grade inflammation has long been recognized as a driver of malignancy through mechanisms involving oxidative DNA damage, immune evasion, and angiogenesis (38). In the context of ESCC, persistent esophagitis—frequently attributable to alcohol and tobacco exposure—may establish a pro-inflammatory microenvironment that facilitates the expansion of neoplastic clones. What we find particularly noteworthy is that, within the subset of 192 recurrent patients, the association between elevated WBC and early recurrence was even more pronounced (OR = 1.649, 95% CI: 1.370–1.986), hinting that systemic inflammation might preferentially accelerate the pace of clonal outgrowth and clinical detection. This interpretation gains support from mechanistic evidence: interleukin-6, a key pro-inflammatory cytokine, activates the JAK/STAT3 and MAPK/ERK signaling cascades, both of which promote tumor cell proliferation and survival (27). Our own *in vitro* experiments further corroborate this notion, as IL-6 stimulation significantly enhanced the viability, proliferation, migratory capacity, and invasive potential of KYSE-30 esophageal squamous carcinoma cells. We should, however, acknowledge that the *in vitro* findings derive from a single cell line under artificial culture conditions, and the extent to which these observations translate to the complex *in vivo* inflammatory milieu remains uncertain; future studies incorporating primary patient samples or animal models would be valuable to validate this mechanistic link.

### 4.3 Macrocytosis and recurrence timing: A dual-layer interpretation

MCV ≥ 106 fL demonstrated an intriguing pattern across our two analytical layers. In the primary Cox analysis using the parsimonious model, MCV ≥ 106 fL remained independently associated with metachronous recurrence (HR: 1.757, 95% CI: 1.144–2.700, *P* = 0.010), consistent with Katada et al. (31) (RR: 2.23).

We interpret this divergence as follows: macrocytosis—a marker of chronic alcohol consumption and/or folate/vitamin B₁₂ deficiency—may not independently determine whether a patient develops metachronous ESCC (as this is primarily driven by the extent of field cancerization), but it may influence how quickly such lesions develop by creating a metabolic environment that facilitates clonal expansion and malignant progression. In the restricted population of recurrent patients (where Lugol C/D was nearly universal at 84.4%), the discriminatory power of endoscopic features was exhausted, allowing MCV's effect to emerge.

This interpretation is preliminarily supported by our exploratory *in vitro* experiments (Figure 8), which demonstrated that folate deficiency alone enhanced KYSE-30 proliferation, migration, and invasion, and that the combination of IL-6 and folate deficiency produced synergistic effects. Folate deficiency disrupts DNA methylation and nucleotide synthesis, fostering genomic instability (25), while inflammatory signaling promotes cell survival (27). Their convergence may accelerate the progression of pre-existing dysplastic clones to clinically detectable carcinoma. We therefore propose a dual-layer model: endoscopic features drive overall risk, while host inflammatory and metabolic factors modulate the tempo of recurrence. This hypothesis requires prospective validation.

### 4.4 Other factors: RBC, BMI, and the male Sex effect

We observed that higher RBC count (HR = 0.344, 95% CI: 0.241–0.492) and higher BMI (HR = 0.942, 95% CI: 0.903–0.982) were each associated with a reduced hazard of metachronous ESCC. One plausible explanation for the RBC finding is that preserved erythrocyte production may signal the absence of alcohol-induced bone marrow suppression and adequate nutritional status, both of which are common in heavy drinkers (31, 39). Regarding BMI, the inverse association we detected aligns with prior reports from Asian populations (40); yet we suspect this relationship may be largely confounded by lifestyle factors, since higher BMI often correlates with better nutrition and less frequent heavy alcohol consumption, rather than reflecting a direct tumor-suppressive effect of adiposity.

In our parsimonious model, male sex was not significantly associated with metachronous recurrence risk (HR: 0.725, 95% CI: 0.511–1.028, *P* = 0.071). Given the wide confidence interval, limited statistical power in the female subgroup (*n* = 451, 44 events), and strong collinearity with lifestyle factors, this estimate is statistically unstable and should not be interpreted as a protective effect.

### 4.5 Smoking and alcohol: confounding and interactions

Although smoking did not retain statistical significance in the parsimonious model (*P* > 0.05 in univariate analysis), its potential role as a confounder was accounted for in the variable selection process. The absence of smoking in the final model may reflect that its effect is largely mediated through endoscopic manifestations of field cancerization, such as Lugol-voiding lesions, a finding that aligns well with the meta-analysis reported by Du et al. (23). We further explored whether the prognostic impact of endoscopic features might differ according to smoking status, and observed a significant interaction between Lugol pattern and smoking (*P*_interaction=0.049), with the hazard ratio for high-risk Lugol pattern being substantially larger among non-smokers (HR = 31.16, 95% CI: 13.57–71.56) than among smokers (HR = 14.96, 95% CI: 9.35–23.94). Before drawing firm conclusions from this interaction, however, several caveats must be considered. The *P* value, while nominally significant, is marginal and would not withstand correction for multiple comparisons (e.g., Bonferroni adjustment), and the number of recurrent events in the non-smoking stratum was relatively small (*n* = 43). We therefore regard this interaction as hypothesis-generating rather than definitive, and we emphasize that independent confirmation in larger, adequately powered cohorts is essential.

Turning to alcohol consumption, we found that this variable failed to show an independent association in the multivariate model. This absence of a direct effect does not necessarily imply that alcohol plays no role in metachronous ESCC; rather, we suspect that endoscopic signs of alcohol-induced field cancerization, such as Lugol-voiding lesions, may capture the cumulative biological impact of alcohol exposure more faithfully than self-reported intake, which is inherently vulnerable to recall bias and imprecise quantification. Furthermore, individual susceptibility to alcohol-related carcinogenesis is strongly modulated by genetic polymorphisms in alcohol-metabolizing enzymes, particularly ALDH2 (rs671) and ADH1B (rs1229984) (16, 18); carriers of inactive or slow-metabolizing alleles experience substantially higher acetaldehyde accumulation and consequently face elevated ESCC risk at equivalent drinking levels. The absence of genotyping data in the present study represents a notable limitation, and we acknowledge that a more precise delineation of the alcohol-risk relationship will require future investigations incorporating ALDH2/ADH1B genotyping and acetaldehyde breath testing (41) to disentangle the interplay between alcohol, genetic susceptibility, and metachronous risk.

### 4.6 Comparison with recent literature

Personalized surveillance after endoscopic resection for superficial ESCC has drawn increasing attention, yet the question remains: can we reliably stratify not only the overall likelihood of metachronous recurrence but also the tempo at which it may manifest? Recent contributions have advanced this field in important ways. Dobashi et al. (32) established a dose-dependent relationship between the severity of Lugol-voiding lesions and cumulative recurrence risk, while Naito et al. (33) proposed a simplified clinical score that integrates endoscopic findings with lifestyle factors. Building on these efforts, our model extends the current framework by incorporating hematological parameters (white blood cell and red blood cell counts) and by offering a refined assessment of margin morphology. Notably, Ishihara (34) called for personalized surveillance strategies; our dual-layer approach directly responds to this call by distinguishing between the baseline risk of recurrence and the timing of its occurrence, a distinction that, to our knowledge, has not been explicitly emphasized in prior work. We acknowledge, however, that our proposed framework is derived from a retrospective cohort and that the exploratory nature of the timing analysis requires prospective validation before it can be adopted in clinical practice.

### 4.7 Clinical implications and future directions

Our parsimonious model's excellent discrimination (C-index 0.924, 95% CI: 0.907–0.940) supports developing a practical risk-stratification framework. Although the exploratory early vs. late recurrence analysis does not support specific surveillance intervals, patients with high-risk Lugol pattern (type C/D), elevated WBC, and macrocytosis may be considered for more frequent follow-up in future prospective studies. However, we emphasize that any surveillance strategy based on recurrence timing remains hypothesis-generating and requires prospective validation before clinical implementation.

A recent randomized trial (42) showed that endoscopic background mucosal resurfacing with radiofrequency ablation effectively prevented metachronous recurrence in patients with multiple LVLs (0% vs. 53% over 5 years). Future prospective studies could evaluate whether our risk model helps identify candidates most likely to benefit from such prophylactic interventions.

Prospective validation in independent cohorts is essential before clinical translation. Incorporating ALDH2/ADH1B genotyping, acetaldehyde breath testing (41), and AI-enhanced endoscopy could further refine risk prediction. Chemopreventive agents (COX-2 inhibitors, statins, metformin) deserve exploration in high-risk populations.

### 4.8 Limitations

Several limitations warrant consideration. The retrospective design introduces inherent bias. ALDH2/ADH1B genotyping was not performed. The male predominance (72.6%) limits generalizability to female patients, and the non-significant association between sex and recurrence risk (HR: 0.725, *P* = 0.071) should be interpreted with caution given the limited statistical power. Excluding patients who received additional therapy after non-curative resection may have introduced selection bias. Residual confounding from unmeasured variables cannot be ruled out.

To address potential center-specific biases arising from different enrollment windows and clinical practices between the two participating centers, we performed a center-stratified Cox regression as a sensitivity analysis (Supplementary Table S3). The hazard ratios remained consistent with the primary model, indicating that center-specific biases had minimal impact on our estimates. Nevertheless, the different enrollment windows between the two centers (Hospital A: 2014-2024; Hospital B: 2020-2024) and the small sample size from Hospital B (*n* = 90, 5.5% of the total cohort) remain inherent limitations of the retrospective multicenter design.

Competing risks from non-ESCC mortality were not accounted for, which may slightly overestimate cumulative incidence; however, the low overall mortality during follow-up suggests minimal bias. External validation in independent cohorts is essential—the C-index may be optimistic in the derivation set.

The exploratory early vs. late recurrence analysis has inherent limitations: the 36.7-month cut-off was cohort-specific and not externally validated; the analysis included only 192 patients with 91 events, limiting statistical power; results are hypothesis-generating and require prospective validation before clinical application.

The *in vitro* experiments also have limitations: single cell line (KYSE-30), supra-physiological IL-6 concentration (intended to model local microenvironment), complete folate deprivation (vs. chronic mild deficiency), and lack of *in vivo* confirmation. These experiments are exploratory mechanistic support, not direct clinical validation. We acknowledge that primary esophageal epithelial cells or *in vivo* animal models would provide stronger evidence for the causal relationship between inflammatory/nutritional disturbances and ESCC progression; however, such experiments were beyond the scope of this retrospective study and require dedicated prospective investigations. The *in vitro* findings should therefore be interpreted as hypothesis-generating, providing preliminary biological plausibility for our clinical observations, rather than definitive mechanistic proof.

We performed LASSO regression with 10-fold cross-validation for variable selection to mitigate overfitting risk. The parsimonious model includes 8 variables with an EPV of 24, well above the recommended minimum of 10. Nevertheless, external validation in independent cohorts is essential before clinical application.

### 4.9 Conclusion

In conclusion, this study demonstrates that endoscopic features—particularly Lugol pattern—are the dominant predictors of overall metachronous recurrence risk after ESD for superficial ESCC. The parsimonious model incorporating eight variables (Age, Sex, BMI, RBC, MCV ≥ 106 fL, WBC, Size, and Lugol_binary) shows excellent discrimination (C-index = 0.924, 95% CI: 0.907–0.940). Exploratory analysis suggests that systemic inflammation (WBC) and nutritional/metabolic disturbance (macrocytosis) may influence recurrence timing, but these findings are hypothesis-generating and require prospective validation. Future studies incorporating genotyping, competing-risk analysis, and AI-enhanced endoscopy may further refine risk prediction.

## Data Availability

The raw data supporting the conclusions of this article will be made available by the authors, without undue reservation.
