# Supplementary material for: Risk factors for metachronous esophageal squamous cell carcinoma after endoscopic submucosal dissection: a multicenter cohort study integrating overall risk prediction and timing of recurrence
Source: Front Med (Lausanne). 2026 Sep 23;13:1952563. doi: 10.3389/fmed.2026.1952563 (PMC13642773; doi:10.3389/fmed.2026.1952563)
Supplement: Supplementary file 1 [file Image1.pdf]

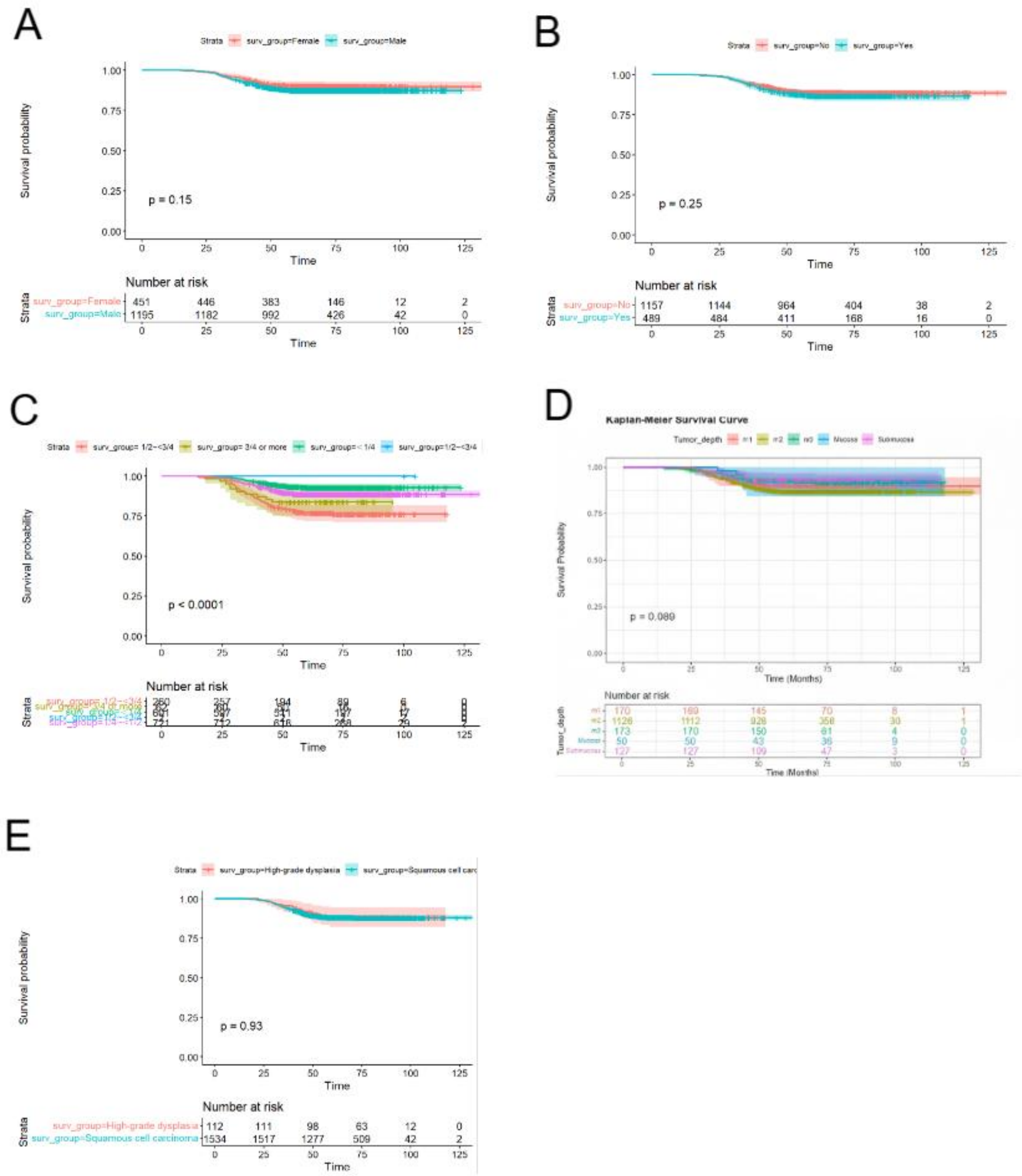

Supplementary Figure S1. Additional Kaplan-Meier curves for metachronous recurrence-free survival.

(A) Stratified by sex (Male, n=1,195; Female, n=451). No significant difference was observed (P=0.147). Medians were not reached in either group.

(B) Stratified by alcohol drinking (No, n=1,157; Yes, n=489). No significant difference was observed (P=0.253). Medians were not reached in either group.

(C) Stratified by circumferential extent (<1/4, n=601; 1/4–<1/2, n=721; 1/2–<3/4, n=260; ≥3/4, n=62). Significant differences were detected (P<0.001). Medians were not reached for any subgroup.

(D) Stratified by depth of invasion (m1, n=170; m2, n=1126; m3, n=173; Mucosa, n=50; Submucosa, n=127). No significant difference was observed (P=0.089). Medians were not reached for any subgroup.

(E) Stratified by final pathology (High-grade dysplasia, n=112; Squamous cell carcinoma, n=1,534). No significant difference was observed (P=0.928). Medians were not reached in either

group.

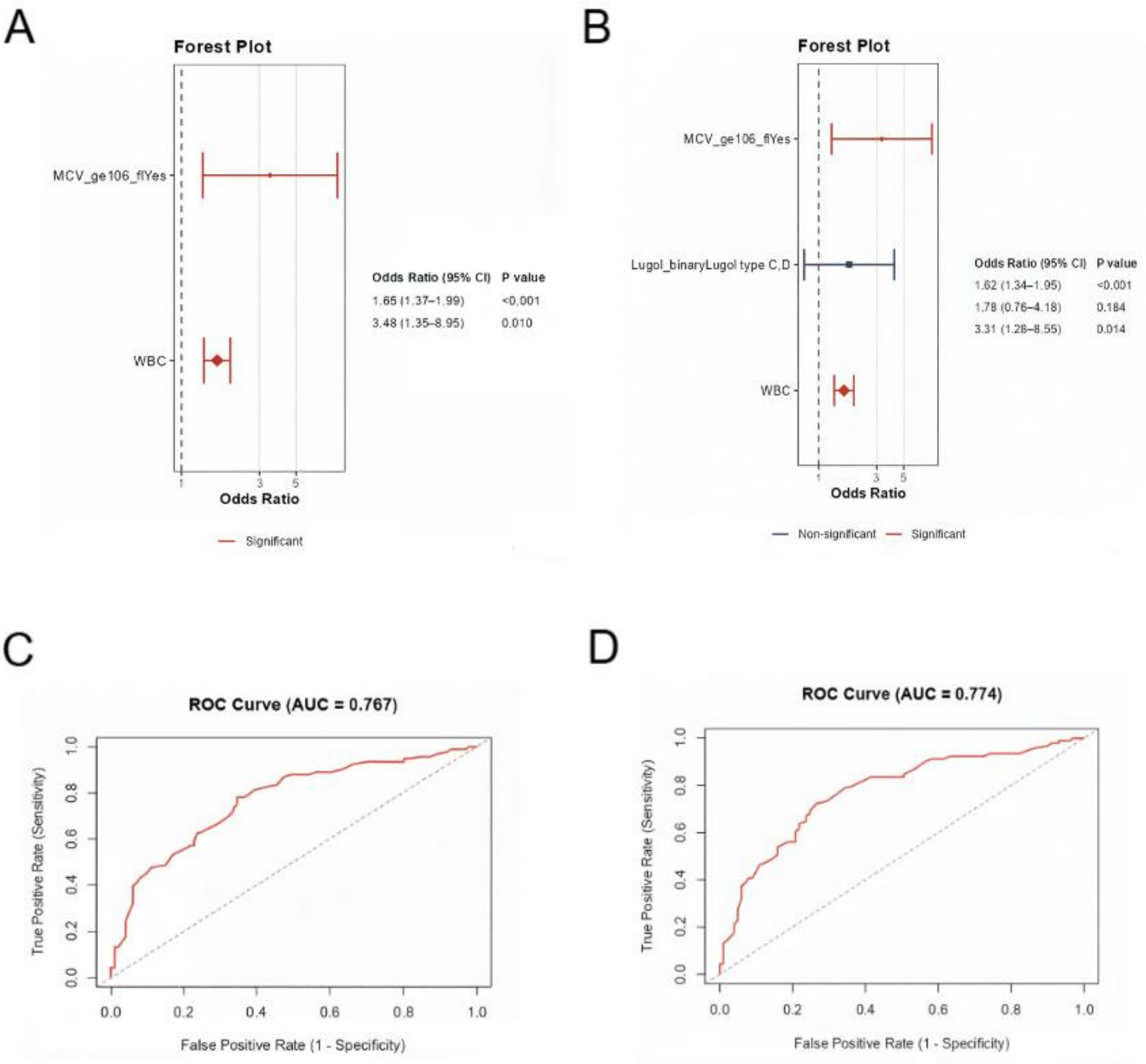

Supplementary Figure S2. Multivariate logistic regression model results.

(A) Forest plot comparing odds ratios (OR) and 95% confidence intervals for the two-variable model (WBC + MCV) (B) Forest plot comparing odds ratios (OR) and 95% confidence intervals for the three-variable model (WBC + MCV + Lugol_binary). (C) ROC curves comparing the two-variable model (AUC=0.767, 95% CI: 0.70-0.83) (D) ROC curves comparing the three-variable model (AUC=0.774, 95% CI: 0.71-0.84). DeLong test *P*=0.682.

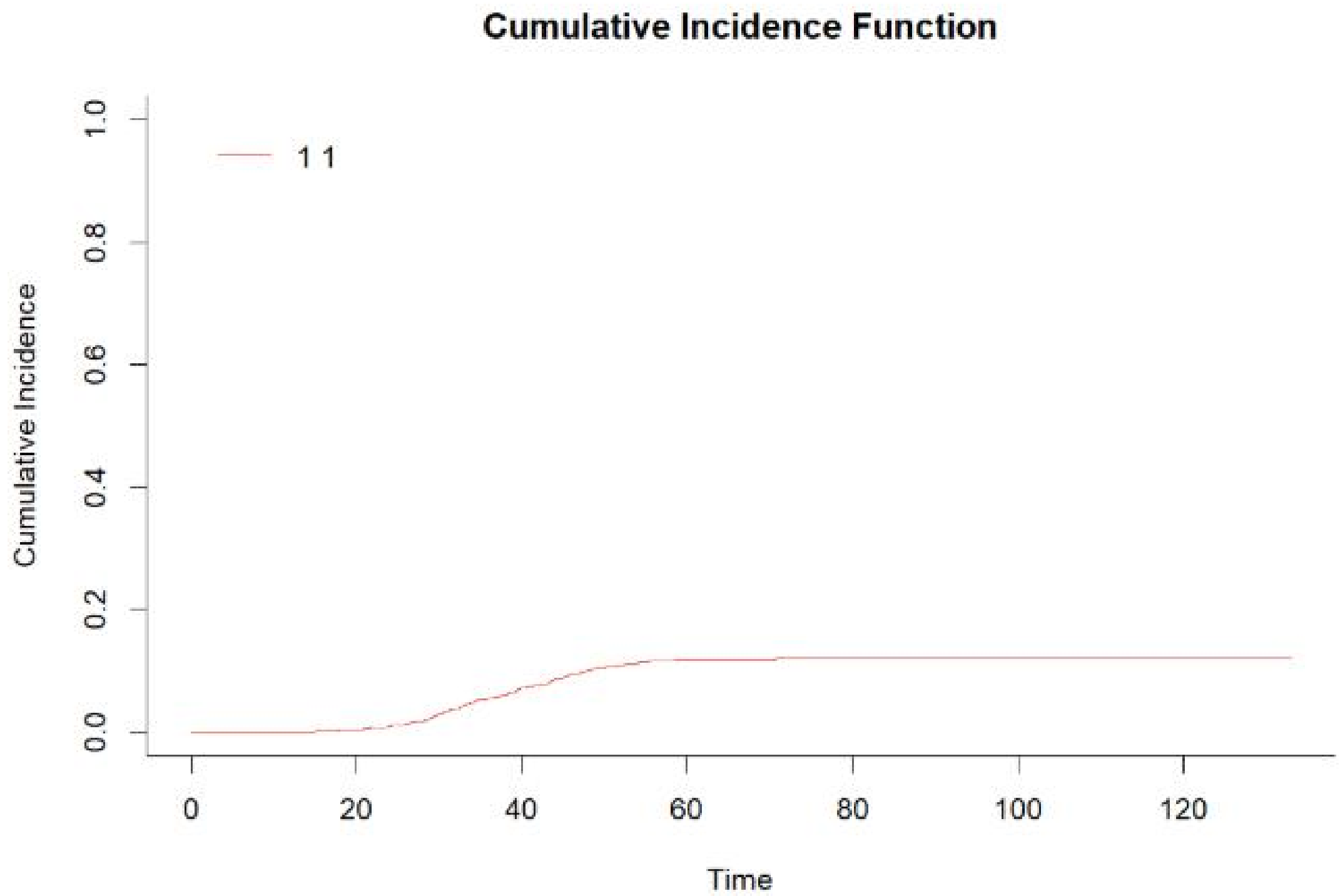

Supplementary Figure S3. Cumulative incidence function (CIF) for metachronous recurrence.

The 5-year cumulative incidence of metachronous recurrence was 11.8% (95% CI: 10.2–13.3%).

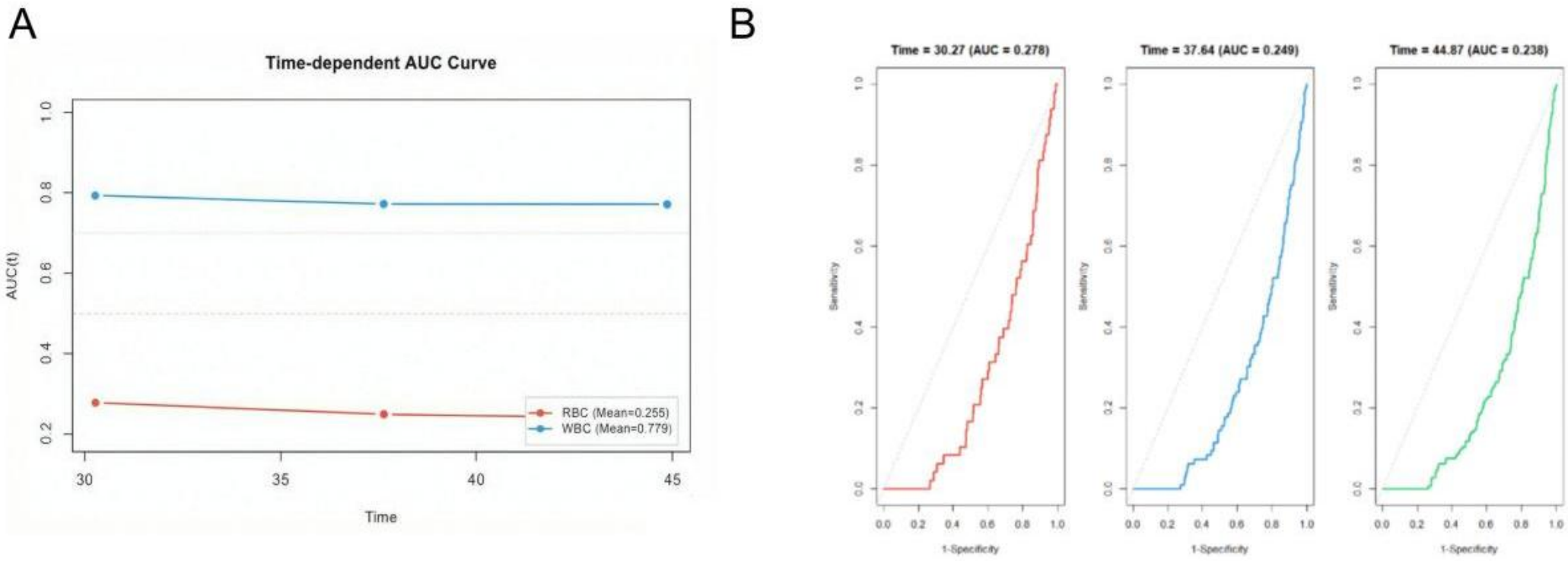

Supplementary Figure S4. Time-dependent ROC analysis for WBC and RBC.

(A) Time-dependent AUC curves showing sustained predictive performance of WBC (iAUC=0.779). (B) ROC curves at 30.27, 37.64, and 44.87 months.
