# Supplementary material for: Risk factors for metachronous esophageal squamous cell carcinoma after endoscopic submucosal dissection: a multicenter cohort study integrating overall risk prediction and timing of recurrence
Source: Front Med (Lausanne). 2026 Sep 23;13:1952563. doi: 10.3389/fmed.2026.1952563 (PMC13642773; doi:10.3389/fmed.2026.1952563)
Supplement: Supplementary file 2 [file Table1.pdf]

Supplementary Table S1. LASSO-selected variables and coefficients

LASSO regression with 10-fold cross-validation identified four predictors with non-zero coefficients (λ = 0.003989).

Variable	Coefficient (β)	OR (exp(β))	Direction
RBC	-2.0046	0.1347	↓ Protective
WBC	0.6077	1.8362	↑ Risk
Size	0.0352	1.0358	↑ Risk
BMI	-0.0247	0.9756	↓ Protective

Supplementary Table S2. Fine-Gray competing risks regression analysis

Variable	sHR	95% CI	P value
Age (per year)	0.994	0.977–1.011	0.490
Sex (Male vs. Female)	0.725	0.490–1.072	0.110
BMI (per kg/m^2)	0.942	0.896–0.989	0.017
RBC (per $10^{12}/L$)	0.344	0.239–0.496	<0.001
MCV ≥106 fL (Yes vs. No)	1.757	1.204–2.565	0.004
WBC (per $10^9/L$)	1.487	1.354–1.634	<0.001
Size (per mm)	1.021	1.012–1.030	<0.001
Lugol type C/D (vs. A/B)	18.868	12.239–29.088	<0.001

Abbreviations: sHR, subdistribution hazard ratio; CI, confidence interval; RBC, red blood cell; WBC, white blood cell; MCV, mean corpuscular volume.

Model performance: Pseudo likelihood ratio test $\chi^2 = 642$, $df = 8$, $P < 0.001$. The Fine-Gray model was fitted using the cmprsk package in R, with non-ESCC mortality treated as the competing event.

Supplementary Table S3. Center-stratified Cox regression analysis

Cox proportional hazards model stratified by center (Hospital A vs. Hospital B), allowing baseline hazards to differ between centers while constraining coefficients for all predictors to be equal.

Variable	HR	95% CI	*P* value
Age (per year)	0.994	0.975–1.013	0.518
Sex (Male vs. Female)	0.726	0.510–1.032	0.073
BMI (per kg/m²)	0.941	0.902–0.981	0.004
RBC (per 10^{12}/L)	0.343	0.240–0.490	<0.001
MCV ≥106 fL (Yes vs. No)	1.758	1.141–2.708	0.011
WBC (per 10^{9}/L)	1.488	1.367–1.619	<0.001
Size (per mm)	1.021	1.011–1.031	<0.001
Lugol type C/D (vs. A/B)	18.872	12.489–28.512	<0.001

Model performance: Harrell's C-index = 0.923 (95% CI: 0.906–0.939); Likelihood ratio test χ^2 *= 641.5, df = 8, P < 0.001.*

Abbreviations: HR, hazard ratio; CI, confidence interval; RBC, red blood cell; WBC, white blood cell; MCV, mean corpuscular volume.

Interpretive note: The hazard ratios from the center-stratified model were nearly identical to those from the primary Cox model (Table 5), confirming that center-specific biases did not materially affect the estimates. The small difference in sample size between centers (Hospital A: n=1,556; Hospital B: n=90) limited the statistical power for detecting center-specific effects.

Supplementary Table S4. Number at risk for Kaplan-Meier curves

Complete data for the "Number at risk" tables presented beneath each Kaplan-Meier curve in Figure 4.

Time point	Overall	Lugol A/B	Lugol C/D	Clear margin	Spiculated margin	Unclear margin	Non-smokers	Smokers	MCV<106	MCV≥106
12 months	1646	1322	324	1114	311	221	473	1173	1575	71
24 months	1631	1310	321	1104	308	219	468	1163	1561	70
36 months	1557	1250	307	1054	294	209	447	1110	1491	66
60 months	1130	907	223	765	213	152	324	806	1082	48

Abbreviations: MCV, mean corpuscular volume.

Source: Data extracted from Kaplan-Meier survival analyses for each subgroup.
